# Radiation inducible MafB gene is required for thymic regeneration

**DOI:** 10.1038/s41598-021-89836-7

**Published:** 2021-05-17

**Authors:** Daiki Hashimoto, Jose Gabriel R. Colet, Aki Murashima, Kota Fujimoto, Yuko Ueda, Kentaro Suzuki, Taiju Hyuga, Hiroaki Hemmi, Tsuneyasu Kaisho, Satoru Takahashi, Yousuke Takahama, Gen Yamada

**Affiliations:** 1 Department of Developmental Genetics, Institute of Advanced Medicine, Wakayama Medical University, Kimiidera 811-1, Wakayama City, Wakayama 641-8509 Japan; 2 Experimental Therapeutics Laboratory, University of South Australia Cancer Research Institute, Clinical and Health Sciences, University of South Australia, Adelaide, SA Australia; 3 Department of Anatomy, Iwate Medical University, Yahaba, Iwate Japan; 4 Department of Urology, Wakayama Medical University, Wakayama, Japan; 5 Laboratory of Immunology, Faculty of Veterinary Medicine, Okayama University of Science, Imabari, Ehime Japan; 6 Department of Immunology, Institute of Advanced Medicine, Wakayama Medical University, Kimiidera, Wakayama Japan; 7 Department of Anatomy and Embryology, Faculty of Medicine, University of Tsukuba, Tennodai, Japan; 8 Experimental Immunology Branch, National Cancer Institute, NIH, Bethesda, MD 20892 USA

**Keywords:** Immunology, Pathogenesis

## Abstract

The thymus facilitates mature T cell production by providing a suitable stromal microenvironment. This microenvironment is impaired by radiation and aging which lead to immune system disturbances known as thymic involution. Young adult thymus shows thymic recovery after such involution. Although various genes have been reported for thymocytes and thymic epithelial cells in such processes, the roles of stromal transcription factors in these remain incompletely understood. MafB (v-maf musculoaponeurotic fibrosarcoma oncogene homolog B) is a transcription factor expressed in thymic stroma and its expression was induced a day after radiation exposure. Hence, the roles of mesenchymal MafB in the process of thymic regeneration offers an intriguing research topic also for radiation biology. The current study investigated whether MafB plays roles in the adult thymus. *MafB*/green fluorescent protein knock-in mutant (*MafB*^+*/*GFP^) mice showed impaired thymic regeneration after the sublethal irradiation, judged by reduced thymus size, total thymocyte number and medullary complexity. Furthermore, IL4 was induced after irradiation and such induction was reduced in mutant mice. The mutants also displayed signs of accelerated age-related thymic involution. Altogether, these results suggest possible functions of MafB in the processes of thymic recovery after irradiation, and maintenance during aging.

## Introduction

The thymus is a primary lymphoid organ that facilitates the differentiation and maturation of lymphoid progenitors into mature naïve T cells^1,2^. Thymus undergoes radiation induced and age-related atrophy, termed thymic involution^3–5^. Such situations commonly arise after clinically induced depletion of immune cells, which is often the case for radiation therapy^6^. Radiation induced tissue damages are considered as one of the essential topics for tissue regeneration studies and radiation biology. Radiation treatment has been demonstrated to severely affect tissue size, cellularity and histology^7,8^. Radiation exposure induces massive apoptosis in stromal cells and most of the thymocytes populations and thymic epithelial cells (TECs)^4,9^. Radiation induced tissue damages are studied for many signaling pathways including reactive oxygen species (ROS)^10–12^. Besides the ROS pathway, cellular interaction such as mesenchyme-TECs, thymocytes and their mediators remain not understood. In a previous study, thymic size and the number of medullary regions (islets) were reported to be reduced after total body irradiation (TBI)^13,14^ and the irradiated mice recovered in some conditions. Sublethal total body irradiation (SL-TBI) is another well-established method for depleting immature thymocyte subsets and disrupting thymic architecture^15^. It is known as an effective model system for analyzing thymic regenerative capacity^16–18^. Although a number of genes have been shown to be essential during thymic recovery after SL-TBI, the roles of stromal transcription factors in this process remain incompletely understood.

Thymus is divided into two major histological regions, the cortex and the medulla, which are separated by a border region termed the cortico-medullary junction (CMJ). The adult thymus is mainly composed of developing lymphocytes (thymocytes) that are supported by a complex three-dimensional network of stromal cells, which include epithelial and mesenchymal cells^19,20^. Adult thymic stroma also includes vascular endothelial cells, macrophages, and monocytes^19^. Such stromal components form unique microenvironments promoting the efficient production of mature naïve T cells^21–23^. Hence, the maintenance of thymic stromal organization and functions is crucial throughout adulthood, particularly during aging and damage-induced (radiation) recovery^24,25^. Radiation induced damages result in the decline of thymic size and total thymocyte number^26^ and it also involves the progressive loss of thymic architecture, which can affect the efficiency of T cell production. In the case of age-dependent thymic changes, the alterations include the reduction of medullary complexity and the loss of distinction between cortex and medulla regions^26–29^. Altogether, these histological changes are established signs of age-related thymic involution^30,31^. Therefore, thymic regenerative capacity is strongly correlated with physiologic age and normal thymus functions for immune competence^30^.

MafB (v-maf musculoaponeurotic fibrosarcoma oncogene homolog B) is a member of the Maf family of transcription factors, characterized by conserved basic region/leucine zipper (bZIP) domains and acidic N-terminal activation domains^32,33^. The bZIP domain mediates dimerization and DNA binding to Maf recognition elements (MAREs), whereas the acidic N-terminal domain facilitates transcriptional activation^34,35^. The expression of AP-1 including MafB is induced by radiation and AP-1 plays essential roles for the early responses after irradiation^36^. In a previous report, MafB has been shown to play a role in embryonic thymus development by regulating lymphocyte accumulation^37^. Such study demonstrated MafB expression in embryonic thymus mesenchyme, and revealed transient lymphocyte accumulation defects during thymus development in MafB-deficient mice. Stromal transcription factors, such as forkhead box N1 (Foxn1), have already been shown by previous studies to be required for both embryonic and adult thymus development^38,39^. Several studies have also demonstrated that thymic stromal cells are essential for the development, maintenance and recovery of adult thymic functions, particularly after radiation-induced damage^16–19^. The current study therefore investigated whether mesenchymal MafB also plays vital roles in the adult thymus.

To examine the roles of MafB in the adult thymus, we utilized *mafB*/green fluorescent protein (GFP) knock-in mutant mice (*MafB*^+*/*GFP^)^40^. Through the SL-TBI model of thymic injury, recovering capacity and maintenance of the thymus was examined by morphological and histological parameters. Alteration of gene expression status was also examined after irradiation. Of note is the induction of *MafB* and essential immune response regulatory cytokine, *IL4*, after SL-TBI. The possible roles of MafB as AP-1 superfamily in such inductive conditions are mentioned. Such histological and q-PCR analyses indicate a possibility of the alteration of immature thymic epithelial progenitor cells (TEPCs) in mutant mice. The characteristics and distribution of MafB-expressing cells in the adult thymus were also investigated. Altogether, these results indicate an intriguing possibility that MafB could be involved in recovery processes from the thymic damage by SL-TBI.

## Results

### Induction of MafB gene expression after sublethal total body irradiation (SL-TBI)

**Figure 1:**
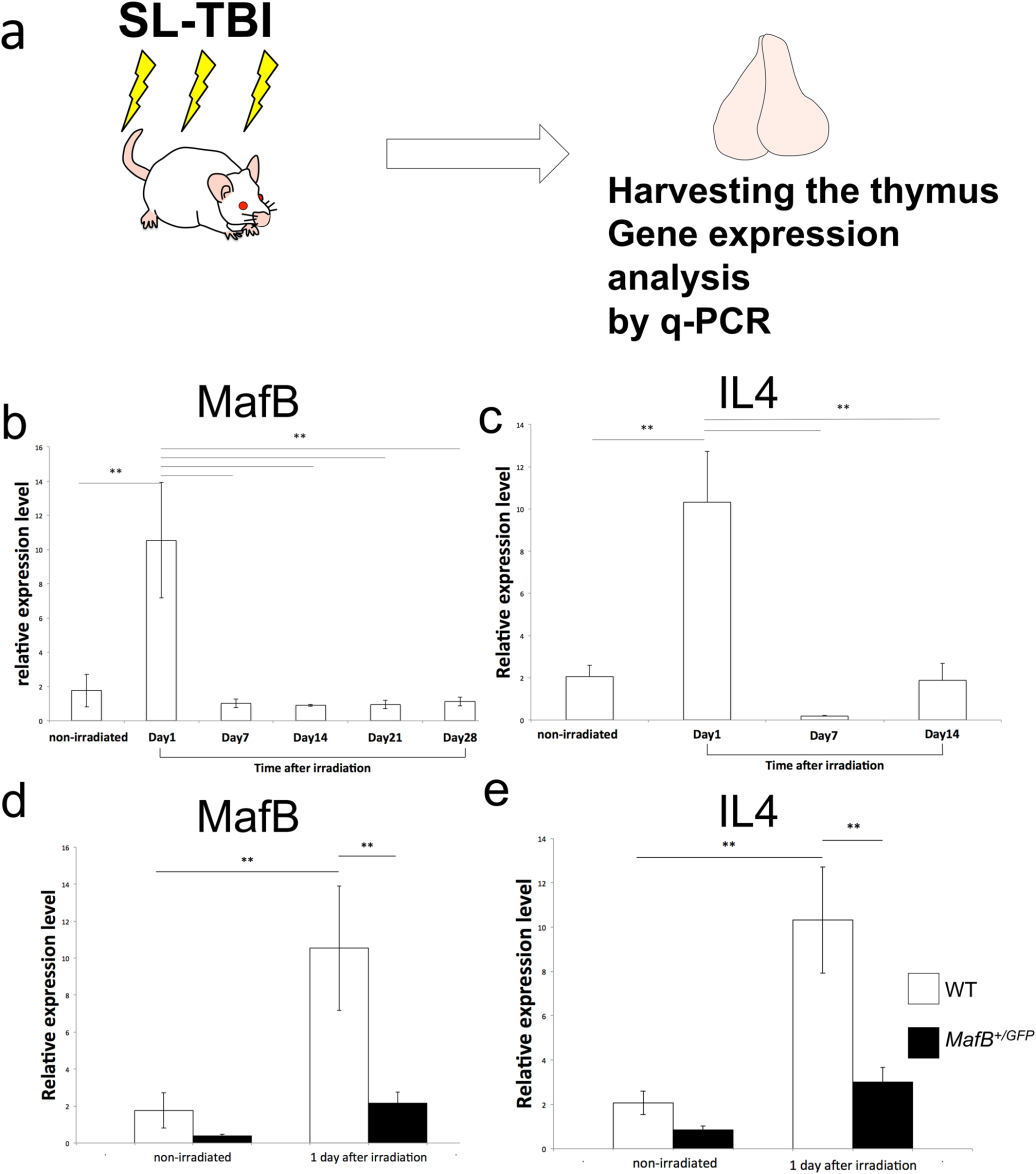
The induction of radiation induced genes, MafB and IL4, in the thymus. (**a**) The schematic illustration indicates the method to examine expression of radiation induced genes. Sublethal total body irradiation (SL-TBI) was performed. Thymic RNA was extracted and q-PCR was performed. (**b**) Prominent MafB gene induction after SL-TBI was detected at Day1 in WT. (**c**) The expression of IL4 after the SL-TBI. Prominent induction of IL4 was detected after 1 day SL-TBI. (**d**) The less prominent induction of MafB expression was reduced in the *MafB*^+*/GFP*^ mutant mice. (**e**) The less prominent of IL4 induction after irradiation in the *MafB*^+*/GFP*^ mutant mice. *P < 0.05, **P < 0.01 (Tukey Kramer method).

It has been known that partial body irradiation induces alteration of gene expression status^36,41,42^. Such expression includes early phase of gene expression and subsequent change of other regulatory gene expression. In order to examine the expression status of MafB gene after SL-TBI, quantitative PCR analyses were performed for the total thymic RNA after the irradiation (Fig.1a). Prominent induction of MafB expression at one day post-SL-TBI was detected (Fig. 1b). Such rapid induction was downregulated at day 7–28 of post-SL-TBI (Fig. 1b). Other examples of radiation induced genes including cytokines were also examined. Induction of radiation inducible cytokine, IL4, was also detected as altered gene expression (Fig. 1c). Such induction was not prominent in the case of irradiated *MafB*^+*/GFP*^ mice compared with WT mice (Fig. 1d, e).

### Thymic MafB-expressing cells were predominantly F4/80 + macrophages/monocytes and ER-TR7 + perivascular fibroblasts associating with CD31+ endothelial compartments

**Figure 2:**
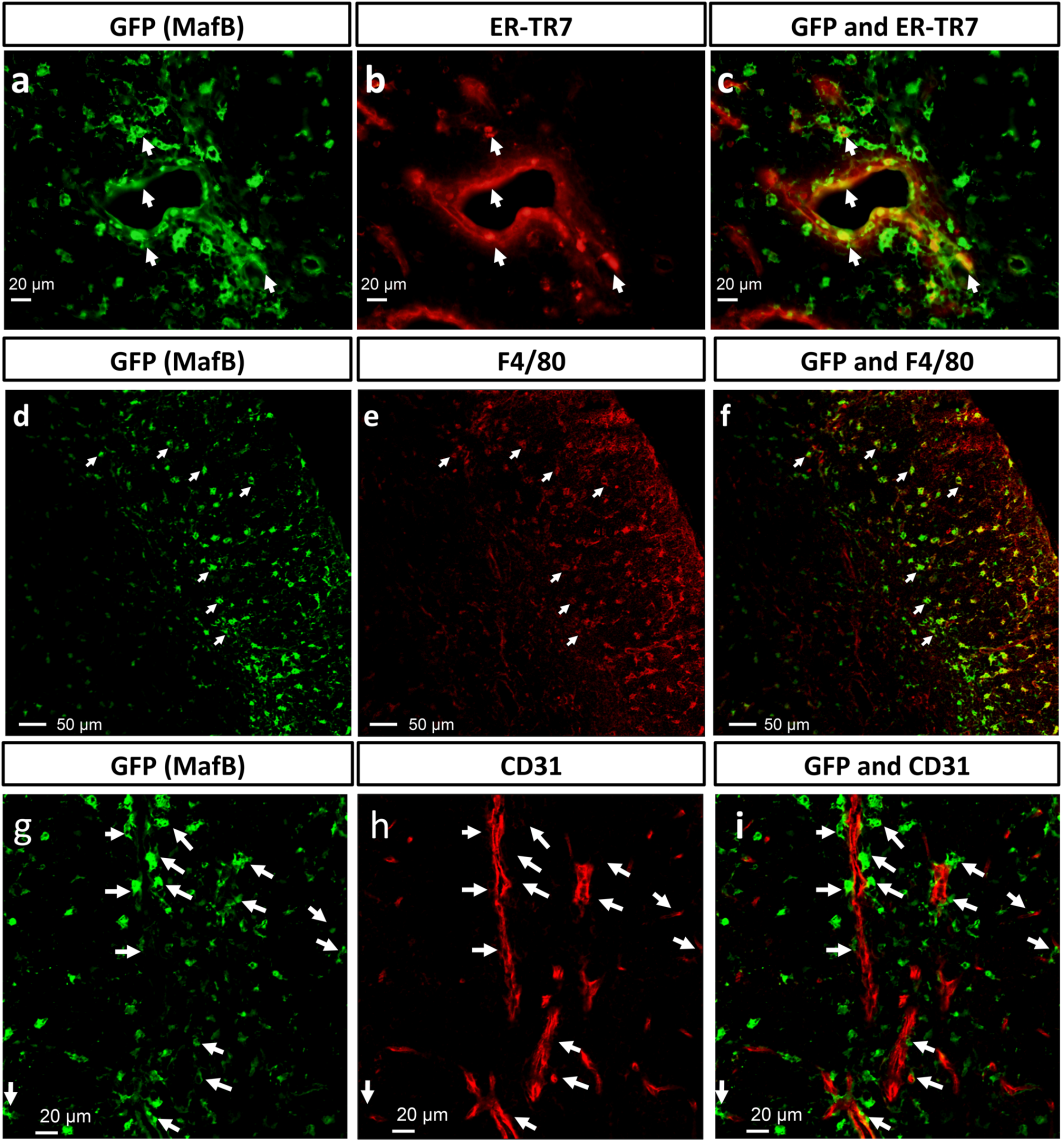
MafB-expressing cells in the adult thymus were predominantly F4/80 + macrophages/monocytes and ER-TR7 + perivascular fibroblasts. (**a**,**b**) GFP expression (green color, representing MafB-expressing cells) and ER-TR7 expression (red color, representing fibroblasts) were shown by immunofluorescence staining of *MafB*^+*/GFP*^ thymus frozen sections (transverse). (**c**) White arrows indicate co-localization of GFP and ER-TR7 expression. Scale bar: 20 µm. (**d**–**e**) GFP expression (green color, MafB-expressing cells) and F4/80 expression (red color, representing macrophages/monocytes) were demonstrated by immunofluorescence staining of *MafB*^+*/GFP*^ thymus frozen sections (transverse). (**f**) White arrows indicate co-localization of GFP and F4/80 expression. Scale bar: 50 µm. (**g**–**h**) GFP expression (green color, representing MafB-expressing cells) and CD31 expression (red color, representing vascular endothelium) shown by immunofluorescence staining of *MafB*^+*/GFP*^ thymus frozen sections (transverse). (**i**) White arrows indicate the localization of GFP positive cells adjacent to endothelial cells. Scale bar: 20 μm. All data shown are representative results of 3 independent experiments using adult specimens from different litters (n = 3).

To identify MafB-expressing cells, immunofluorescence staining was performed using GFP and ER-TR7 (as a marker of fibroblasts) in the adult *MafB*^+*/GFP*^ thymus^43^. Some GFP + cells were positive for ER-TR7 expression and located adjacent to blood vessels, indicating that these cells were ER-TR7 + fibroblasts (Fig. 2a-c; indicated by white arrows). However, a significant number of GFP + cells did not show its expression (data not shown). Conversely, many of the GFP + cells were positive for F4/80, which is a macrophage/monocyte marker (Fig. 2d-f; indicated by white arrows). This indicated that a large portion of MafB-expressing cells in the adult thymus were F4/80 + macrophages/monocytes. Furthermore, most of these GFP + F4/80 + macrophages/monocytes were located in the cortex and along the CMJ region (Fig. 2d-f). A similar tendency was confirmed when immunofluorescence staining was performed on adult WT thymic sections for endogenous MafB and F4/80 expression (data not shown). Altogether, these findings reveal that MafB-expressing cells are predominantly composed of F4/80 + macrophages/monocytes and ER-TR7 + perivascular fibroblasts.

To further investigate the localization of MafB-expressing cells in adult thymic microenvironments, we co-stained GFP with the vascular endothelial cell marker CD31 in adult *MafB*^+*/GFP*^ thymi, and analyzed stromal cell localization by confocal microscopy. Part of the GFP-expressing cell population appeared to locate adjacent to CD31 + vascular endothelium (Fig. 2g-i; white arrowheads). Such images revealed multiple locations where GFP + cells were adjacent to CD31 + blood vessels of various sizes (Fig. 2g-i; white arrows). Such blood vessels included capillaries (diameter ranging from 3.5 to 7 µm) located throughout the adult thymus (Fig. 2), and postcapillary venules (PCVs) (diameter greater than 15 µm) (supplemental Fig. 1a-c; white asterisks), which were usually located along the CMJ region^44,45^. The large blood vessels such as PCVs were often observed adjacent to multiple GFP-expressing cells (Fig. 2a-c). A similar tendency was confirmed when immunofluorescence staining was performed on adult WT thymic sections for endogenous MafB and CD31 expression (data not shown). Taken together, these results demonstrate that many MafB-expressing cells were located adjacent to the perivascular region, often in close proximity to thymic vasculature.

### MafB^+*/GFP*^ mice showed impaired thymic recovery after sublethal total body irradiation (SL-TBI)

To investigate the possible roles of MafB in adult thymus development, the thymi of 9-week-old *MafB*^+*/GFP*^ mice and wild-type (WT) littermates were analyzed based on size, mass and total thymocyte number. *MafB*^+*/GFP*^ and WT mice showed no significant differences by such analyses (Supplementary Fig. 2a-c). These results suggest that young adult *MafB*^+*/GFP*^ mice develop thymi without prominent abnormalities under normal physiological conditions.

**Figure 3:**
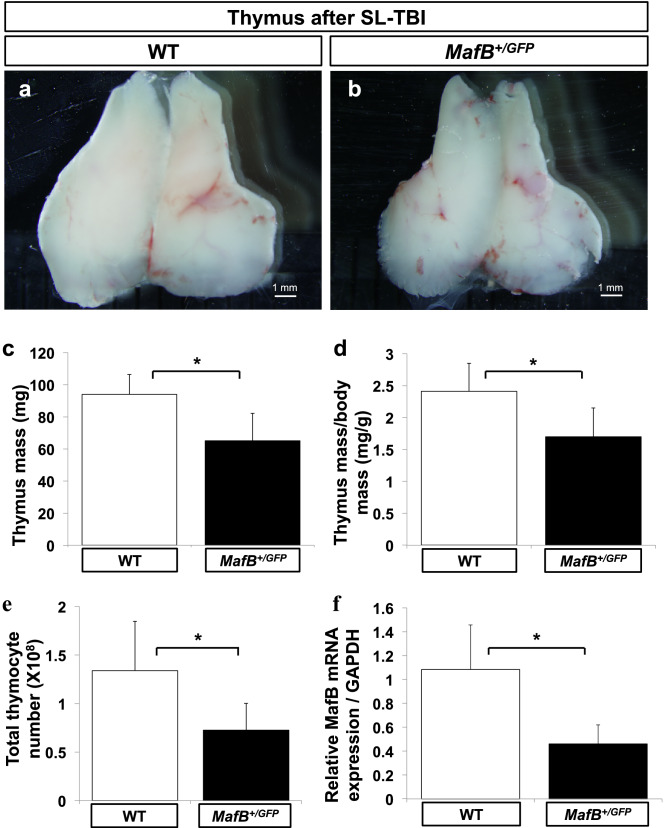
*MafB*^+*/GFP*^ mice showed impaired thymic recovery after sublethal total body irradiation (SL-TBI). (**a**–**e**) Thymi from 9-week-old wild-type (WT) (white bars, n = 6) and *MafB*^+*/GFP*^ (black bars, n = 6) mice were analyzed 28 days after SL-TBI. (**a**,**b**) Gross morphology of thymus. Scale bar: 1 mm. (**c**) Absolute thymus mass (mg; milligrams). (**d**) Normalized mass calculated as thymic mass divided by body mass (mg/g). (**e**) Total thymocyte number, measured using a haemocytometer. All data are shown as the means ± standard deviation (SD). *P < 0.05 (Student’s *t* test). (**f**) The thymi of *MafB*^+*/GFP*^ mice showed reduced *MafB* mRNA expression compared to those of WT counterparts. Total RNA was extracted from whole thymi of untreated 5-week-old WT (white bar, n = 6) and *MafB*^+*/GFP*^ (black bar, n = 6) mice. *MafB* mRNA expression was subsequently measured by qRT-PCR, and normalized to *GAPDH* mRNA expression. Data presented as the means ± SD. *P < 0.05 (Student’s *t* test). All data shown are representative results of at least 3 independent experiments using adult specimens from different litters (n ≥ 3).

Previous studies have shown that introduction of mutation in some stromal genes can result in the impaired recovery and/or maintenance of adult thymi, particularly after radiation-induced damage^19,30,38^. We therefore asked whether MafB may play a role in thymic regeneration after damages. To test this possibility, 5-week-old *MafB*^+*/GFP*^ mice and WT littermates were exposed to SL-TBI. Assessment for thymic recovery was performed 28 days after exposure, which represents the stage of sufficient recovery in young adult thymi^16–18^. The thymi of *MafB*^+*/GFP*^ mice were smaller than those of WT counterparts 28 days after SL-TBI (Fig. 3a, b). This size difference was verified by measuring thymic mass normalized by body mass, and total thymocyte number (Fig. 3c-e). The absolute mass and normalized mass of SL-TBI-treated *MafB*^+*/GFP*^ thymi were approximately 30% less than those of WT counterparts (Fig. 3c, d; 93.9 ± 12.4 mg in WT mice vs. 65.1 ± 17.0 mg in *MafB*^+*/GFP*^ mice; n = 6). Moreover, total thymocyte number of SL-TBI-treated *MafB*^+*/GFP*^ thymi was approximately 45% less than that of WT counterparts (Fig. 3e; 1.34 ± 0.51 × 10^8^ in WT mice vs. 7.26 ± 2.76 × 10^7^ in *MafB*^+*/GFP*^ mice; n = 6). To examine the extent of MafB expression, the expression level of *MafB* mRNA in whole thymi was compared by quantitative reverse transcription-PCR (qRT-PCR) between untreated WT and *MafB*^+*/GFP*^ mice at 5 weeks of age. The thymi of *MafB*^+*/GFP*^ mice showed significantly reduced *MafB* mRNA expression (50 ~ 60% reduction) compared to those of WT counterparts (Fig. 3f; n = 6).

### MafB^+*/GFP*^ mice showed impaired restoration of thymic architecture after SL-TBI

**Figure 4:**
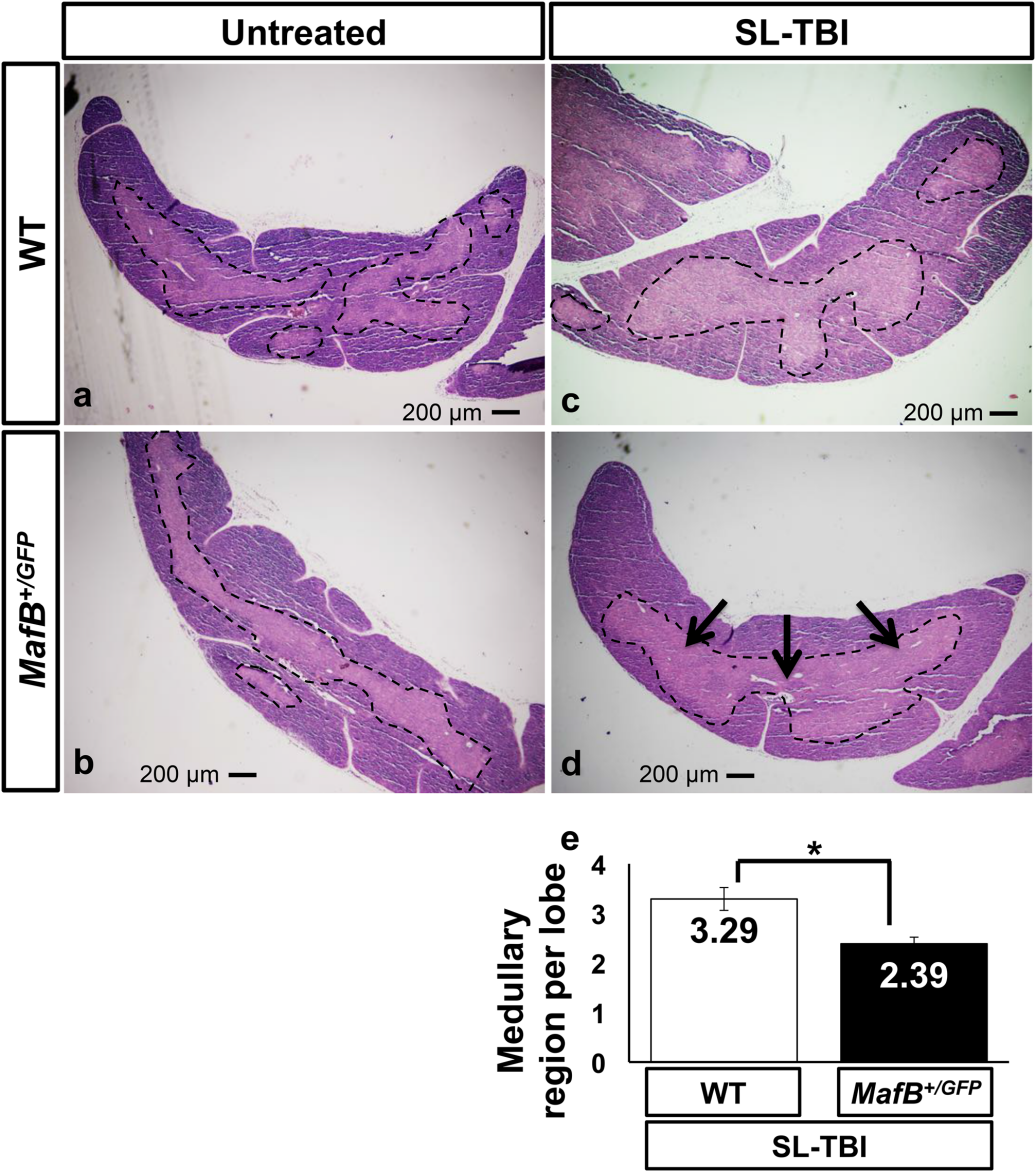
*MafB*^+*/GFP*^ mice showed impaired restoration of thymic architecture after SL-TBI. (**a**–**d**) Hematoxylin and Eosin (H.E.) staining of 9-week-old *MafB*^+*/GFP*^ and WT thymi paraffin serial sections (transverse), 28 days after SL-TBI. The borders of medullary region are indicated by black dotted lines. Scale bar: 200 µm. Multiple medullary region were observed in (**a**) untreated WT thymi, (**b**) untreated *MafB*^+*/GFP*^ thymi, and (**c**) SL-TBI-treated WT thymi. (**d**) SL-TBI-treated *MafB*^+*/GFP*^ thymi showed reduced number of medullary region compared to those of SL-TBI-treated WT littermates. Transverse sections of SL-TBI-treated *MafB*^+*/GFP*^ thymi frequently exhibited a single large medulla region (indicated by black arrows), which was rarely observed in SL-TBI-treated WT counterparts. (**e**) Quantification of medullary region per thymic lobe was performed based on H.E.-stained serial sections (approximately 800 sections) from SL-TBI-treated WT and *MafB*^+*/GFP*^ thymi. Data in (**e**) are shown as the means ± SD. *P < 0.05 (Student’s *t* test). All data shown are representative results of 3 independent experiments using adult specimens from different litters (n = 3).

Total body irradiation is known to disrupt thymic architecture and reduce medullary complexity, which is defined as the number of medullary regions per thymic lobe^14,15,28,46^. In young and healthy mice, thymic recovery after irradiation includes the restoration of thymic architecture and medullary complexity^14,28^. Due to the impaired thymic recovery of *MafB*^+*/GFP*^ mice, we hypothesized that the restoration of thymic architecture after irradiation might also be affected in such mice. To test this hypothesis, serial transverse sections from irradiated and untreated thymi were analyzed after hematoxylin and eosin (H.E.) staining. Thymic medullary regions (islets) were visualized as distinct medullary areas circumscribed by cortical area (Fig. 4a-d; borders of medullary regions indicated by black dotted lines). In untreated mice, the architecture of WT thymi appeared to have slightly more medullary regions than that of *MafB*^+*/GFP*^ thymi (Fig. 4a,b). After quantification of medullary region number, such differences were found to be statistically insignificant (data not shown). In irradiated mice, WT thymus architecture was restored histologically similar to those of untreated counterparts 28 days after SL-TBI (Fig. 4a,c). In contrast, *MafB*^+*/GFP*^ thymi showed impaired restoration of thymic architecture compared to WT counterparts, 28 days after SL-TBI (Fig. 4c,d). Transverse sections of SL-TBI-treated *MafB*^+*/GFP*^ thymi frequently exhibited a single large medulla region (Fig. 4d; indicated by black arrows), which was rarely observed in SL-TBI-treated WT counterparts (Fig. 4c). Such defects were verified quantitatively in terms of medullary complexity, wherein SL-TBI-treated *MafB*^+*/GFP*^ thymi displayed significantly reduced number of medullary regions compared to WT counterparts (Fig. 4e). Moreover, similar results were obtained when sagittal sections of whole thymic lobes were analyzed after H.E. staining (Supplementary Fig. 3a-d).

**Figure 5:**
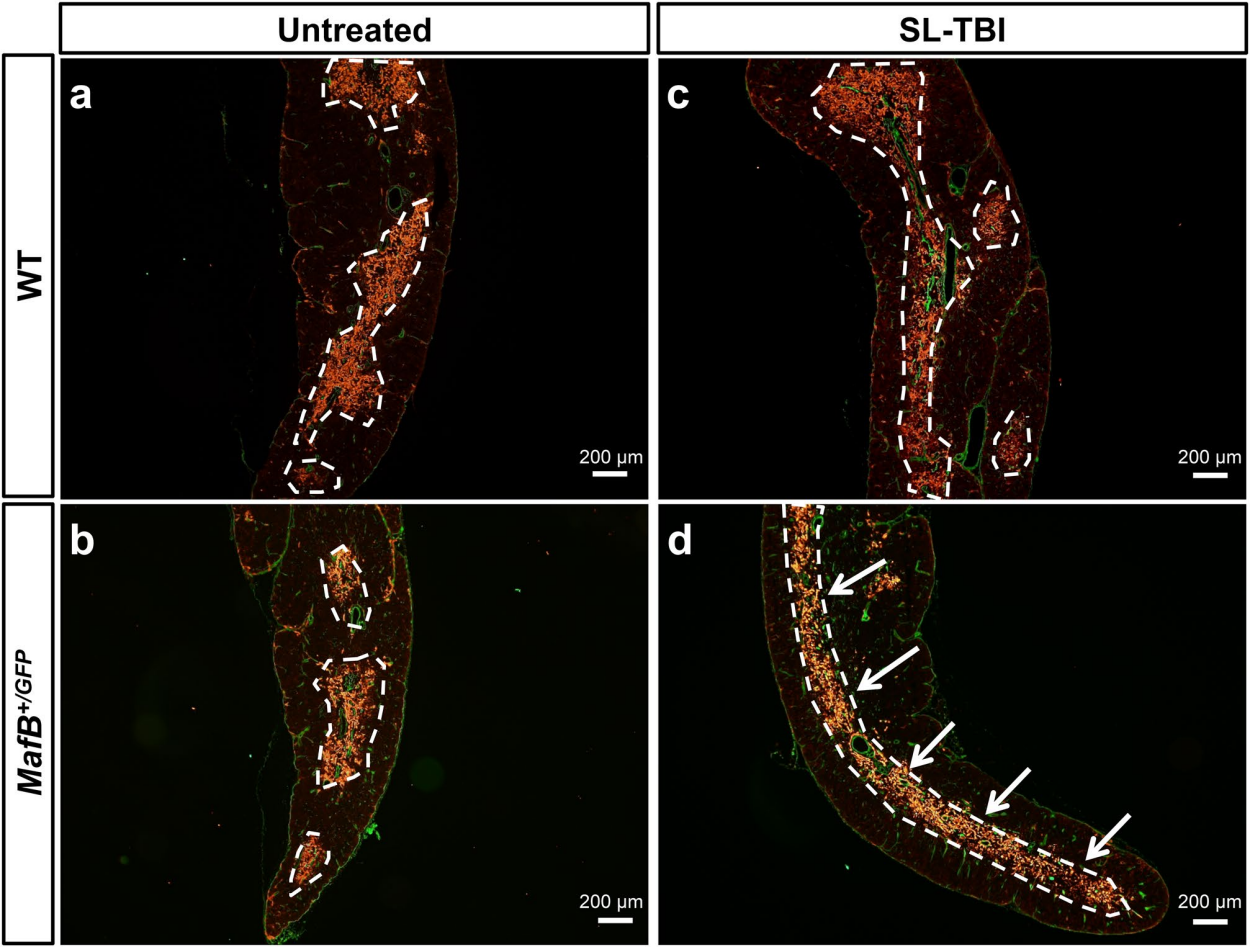
Thymi from *MafB*^+*/GFP*^ mice displayed aberrant organization of medullary thymic epithelial cell (mTEC) clusters after SL-TBI. (**a**–**d**) ER-TR7 expression (green color, representing fibroblasts and other mesenchymal cells of the thymic capsule) and Keratin 14 expression (red color, representing mTECs) shown by immunofluorescence staining of 9-week-old *MafB*^+*/GFP*^ and WT thymi frozen sections (transverse), 28 days after SL-TBI. The borders of Keratin 14 positive mTEC clusters are indicated by white dotted lines. Scale bar: 200 µm. Similar organization of Keratin 14 positive mTEC clusters was observed in (**a**) untreated WT thymi, (**b**) untreated *MafB*^+*/GFP*^ thymi, and (**c**) SL-TBI-treated WT thymi. (**d**) Aberrant organization of mTEC clusters was prominently observed in SL-TBI-treated *MafB*^+*/GFP*^ thymi, which often exhibited a single large mTEC cluster (indicated by white arrows). All data shown are representative results of 3 independent experiments using adult specimens from different litters (n = 3).

In addition to H.E. staining, another established method of defining medullary region is to examine the expression of medullary thymic epithelial cell (mTEC) markers (e.g. Keratin 14)^14,47^. Based on this definition, medullary complexity can be analyzed by the organization of Keratin 14 + mTEC clusters. To examine whether the impaired restoration of thymic architecture could be partly due to altered organization of mTECs, immunofluorescence staining was performed with the mTEC marker Keratin 14^14,47^. The organization of Keratin 14 + mTEC clusters in the thymi of untreated *MafB*^+*/GFP*^ mice appeared similar to that of untreated WT mice, characterized by the presence of multiple distinct mTEC clusters distributed across each tissue area (Fig. 5a,b). The organization of such clusters also appeared similar between untreated and irradiated WT thymi, indicating sufficient restoration after SL-TBI (Fig. 5a,c). In contrast, the Keratin 14 + mTEC clusters in SL-TBI-treated *MafB*^+*/GFP*^ thymi often appeared as a single prominent medullary compartment (Fig. 5d; indicated by white arrows), which was not observed in WT littermates (Fig. 5c). Such aberrant mTEC organization in SL-TBI-treated *MafB*^+*/GFP*^ thymi is consistent with the decreased medullary complexity observed after H.E. staining (Fig. 4d). Altogether, the histological abnormalities evident in SL-TBI-treated *MafB*^+*/GFP*^ thymi indicated impaired restoration of thymic architecture, in terms of reduced number of medullary region and altered histology of the medulla compartment. These results suggest that MafB is required for the restoration of thymic architecture after SL-TBI.

### Less prominent presence of immature TECs with reduced expression of Krt5 and FoxN1 in MafB^*+/GFP*^ thymi after SL-TBI

**Figure 6:**
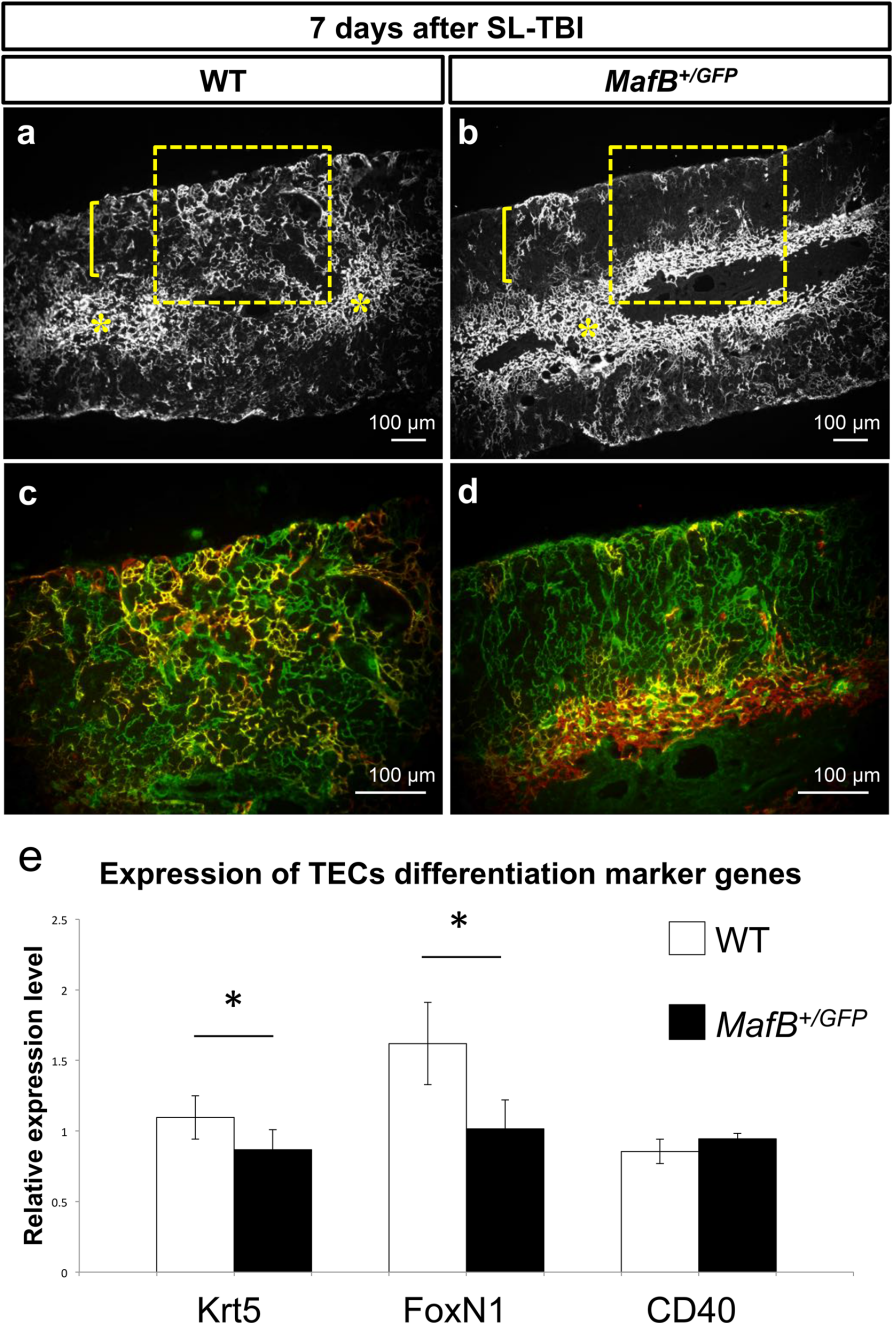
Cortical keratin 5/8 double positive cells were less prominently detected after SL-TBI; abnormal immature TECs marker expression in MafB mutant mice thymi. (**a**–**d**) Immunofluorescence staining of 6-week-old *MafB*^+*/GFP*^ and WT thymi frozen sections (transverse), 7 days after SL-TBI. (**a**,**b**) Low magnification images showing Keratin 5 expression (white color, representing mTECs). Yellow asterisks indicate the medulla regions, whereas yellow brackets indicate cortex regions. Enclosed areas include parts of the cortex, cortico-medullary junction (CMJ) and medulla regions. Scale bar: 100 µm. (**c**,**d**) High magnification images showing both Keratin 8 expression (green color, representing cTECs) and Keratin 5 expression (red color, mTECs) of enclosed areas in (**a**,**b**). Co-localization of Keratin 5 and Keratin 8 expression is indicated by yellow color. Scale bar: 100 µm. (**a**,**b**) Keratin 5 positive cells were detected in the medulla regions of both *MafB*^+*/GFP*^ and WT thymi. Such cells were also present in the cortex regions of WT thymi, but were less apparent in the cortex regions of *MafB*^+*/GFP*^ thymi. (**c**) Cells expressing both Keratin 5 and Keratin 8 were prominently detected in the cortex of WT thymi. (**d**) Keratin 5/8 double positive cells were less prominently detected in the cortex of *MafB*^+*/GFP*^ thymi. All data shown are representative results of 3 independent experiments using adult specimens from different litters (n = 3). (**e**) The expression of TECs differentiation marker genes after irradiation. Krt5 and FoxN1 expressions were reduced in *MafB*^+*/GFP*^ mutant compared with those of WT. CD40 is expressed in mature TECs in controls and such expression was not altered in mutants. *P < 0.05 (Student’s *t* test).

It has previously been suggested that the development and organization of mTECs is partly regulated by thymic epithelial progenitor cells (TEPCs), presumed to reside in the cortex and CMJ regions^46,48–52^. We therefore analyzed TEPCs after irradiation to determine if such cell populations are affected. Because thymic regeneration and TEPC expansion generally initiate within 1 week after irradiation, we investigated the distribution of such progenitors 7 days after SL-TBI. Cells expressing both Keratin 5 and Keratin 8 (Keratin 5/8 double positive cells) were considered to include TEPCs, as suggested by previous studies^48–53^. Keratin 5 positive cells were detected in the medulla regions of both *MafB*^+*/GFP*^ and WT thymi, 7 days after irradiation (Fig. 6a,b; indicated by yellow asterisks). Such cells were also clearly present in the cortex regions of WT thymi, but were less apparent in the cortex regions of *MafB*^+*/GFP*^ thymi (Fig. 6a,b; indicated by yellow brackets and enclosed areas). Furthermore, keratin 5/8 double positive cells were observed along the CMJ and in the cortex regions of SL-TBI-treated WT thymi (Fig. 6c; indicated by yellow color). On the other hand, such double positive cells were observed along the CMJ of SL-TBI-treated *MafB*^+*/GFP*^ thymi, but were less prominently detected in the cortex regions compared to those of WT counterparts (Fig. 6d). These results demonstrate that cortical Keratin 5/8 double positive cells are less prominently detected in *MafB*^+*/GFP*^ thymi compared to WT counterparts 7 days after irradiation.

In order to examine further TEC differentiation status, expression of several TEC marker genes after irradiation were examined by q-PCR analysis. Krt5 and FoxN1 genes are known as immature cell marker and transcription factor respectively^38,48^. Significant reductions of Krt5 and FoxN1 expression were detected by q-PCR analyses in mutants thymi (Fig. 6e). In contrast, the expression of CD40, known as a mature mTEC marker receiving the signals from thymocytes in postnatal period, was not changed (Fig. 6e).

### MafB^+*/GFP*^ mice showed signs of accelerated age-related thymic involution

**Figure 7:**
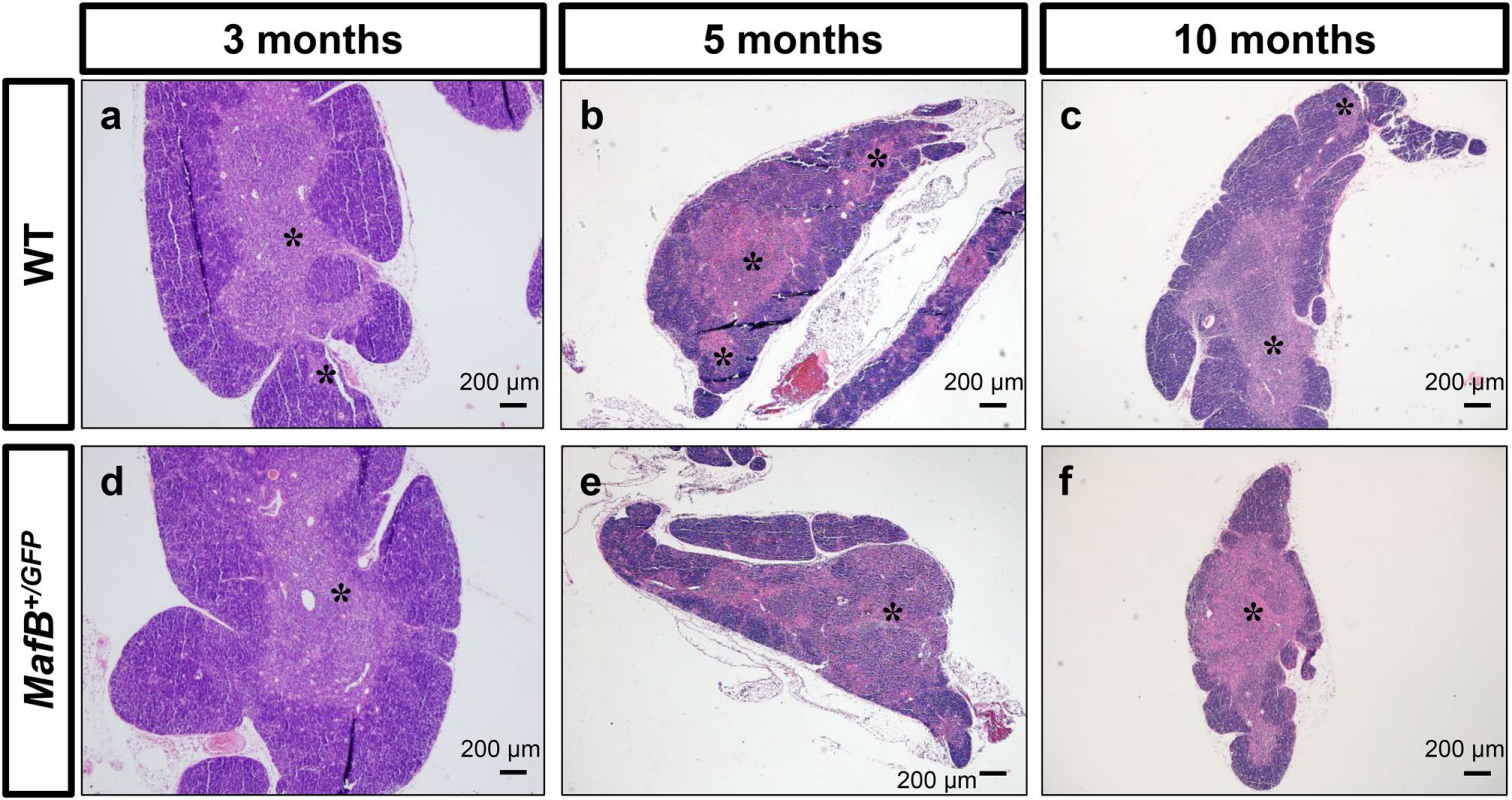
*MafB*^+*/GFP*^ mice showed signs of accelerated age-related thymic involution. (**a**–**f**) Hematoxylin and Eosin (H.E.) staining of 3, 5 and 10-month-old *MafB*^+*/GFP*^ and WT thymi paraffin sections (transverse). Scale bar: 200 µm. (**a**,**b**) 3 and 5-month-old WT thymi showed normal thymic architecture, judged by the presence of medullary region with a clear distinction between cortex (purple-colored regions) and medulla (pink-colored regions). Black asterisks indicate medullary region. (**c**) 10-month-old WT thymi displayed signs of age-related thymic involution, with slightly reduced number of medullary region and slight loss of distinction between cortex and medulla (relatively small intermixed pink and purple regions). (**a**,**d**) The thymi of 3-month-old *MafB*^+*/GFP*^ mice were histologically similar to those of WT littermates. (**b**,**c**,**e**,**f**) The thymi of 5-month-old and 10-month-old *MafB*^+*/GFP*^ mice exhibited slightly decreased number of medullary region compared to those of WT littermates. In contrast to WT counterparts, mutant thymi also displayed a greater loss of distinction between cortex and medulla, wherein a larger portion of the tissue area is occupied by medulla regions (pink) interspersed with cortex regions (purple). These observations suggest that *MafB*^+*/GFP*^ thymi showed signs of accelerated age-related thymic involution compared to WT thymi. All data shown are representative results of 3 independent experiments using adult specimens from different litters (n = 3).

It is well-established that thymic regenerative capacity is correlated with the physiologic age of the thymus^30^. The thymic recovery defects observed in *MafB*^+*/GFP*^ mice might therefore be correlated with thymic maintenance during aging. To examine such possibility, we performed histological analyses of *MafB*^+*/GFP*^ and WT thymi at different adult stages (3, 5 and 10 months old). The thymi of 3-month-old and 5-month-old WT mice showed a clear distinction between cortex (purple-colored regions) and medulla (pink-colored regions) (Fig. 7a,b). In contrast, slightly decreased medullary region number and a mild loss of distinction between cortex and medulla regions were observed in 10-month-old WT thymi (Fig. 7c), compared to 5-month-old WT counterparts (Fig. 7b; medullary region indicated by asterisks). This loss of distinction appears as slight intermixing of the purple-colored regions (cortex) and the pink-colored regions (medulla) (Fig. 7c). Such alterations are consistent with the hallmarks of age-related thymic involution, reported previously^28,29,31^.

The thymi of 3-month-old *MafB*^+*/GFP*^ mice were similar to those of WT littermates, judged by H.E. staining (Fig. 7a,d). But compared to WT littermates, the thymi of 5-month-old *MafB*^+*/GFP*^ mice showed slightly decreased number of medullary region, and loss of distinction between cortex and medulla (Fig. 7b,e; medullary regions indicated by asterisks). The greater loss of distinction between cortex and medulla in mutant thymi resulted in the formation of larger tissue areas where medulla regions (pink) interspersed with cortex regions (purple) (Fig. 7e). Similar histological differences were also observed between the thymi of 10-month-old *MafB*^+*/GFP*^ and WT littermates (Fig. 7c,f).

In addition to histological findings, the absolute and normalized mass of 12-month-old *MafB*^+*/GFP*^ thymi were determined to be 15 ~ 20% less than those of WT counterparts (Supplementary Fig. 4a,b; 33.8 ± 4.4 mg in WT mice vs. 27.0 ± 4.7 mg in *MafB*^+*/GFP*^ mice; n = 5). Altogether, such observations indicate that *MafB*^+*/GFP*^ mice show signs of accelerated age-related thymic involution compared to WT littermates.

## Discussion

Thymus is capable of regeneration which is observed after its irradiation and during aging^30,54^. However, the mechanisms underlying thymic regeneration remain unclear. Particularly, the roles of non-epithelial stromal transcription factors in such processes remain poorly understood. In the current study, the mutant mice for MafB gene (one of the AP-1 superfamily transcription factor genes) displayed both impaired thymic recovery after the SL-TBI, and accelerated age-related involution. We investigated the roles of stromal MafB for thymic radiation induced regeneration and aging in the current study.

### Radiation induced genes and thymic regeneration

It is well known that radiation induces thymic damages including abnormalities of thymocytes^55^. Of note is the massive thymocyte death after the irradiation which leads to the defective immune responses^56^. One of the essential signals involved after irradiation is the pathways including ROS (reactive oxygen species) and they were reported as locating upstream of immediate early response genes. Such genes include AP-1 superfamily which is a group of dimeric transcription factors. AP-1 is activated by various extracellular signals^57^. Such signals include growth factors, hormones, cytokine and other stress signals for cells. Radiation induces several early response genes such as c-jun and c-fos^58^. In fact, AP-1 superfamily is reported as playing essential roles as downstream of ROS signal^59,60^. These early response genes regulate downstream genes that are important for the adaptation of thymus for radiation-induced stress. Among several lymphoid organs, thymus is known as one of the most sensitive organs for radiation induced damages. In the case of young thymus, they show regenerative capability such as after radiations. Thus, studies on the radiation induced regeneration are essential topics for the analysis of early response genes and also in radiation biology.

MafB, which belongs to the AP-1 superfamily of genes, possesses essential roles in male reproductive organ mesenchyme^61^. In such developmental context, it locates underneath of male hormone signals acting on genital mesenchyme^57^. MafB gene is expressed and has functions in the thymus, bone and spleen where radiation sensitivity is reported^62–64^. Recently, it is also reported that MafB gene is involved for the regulation of cell death in limb bud interdigit region^65^. Interdigit region in the limb is known as essential region showing cell death during development^66^. The study suggested MafB gene is essential for such regulation and ROS signal is detected in the interdigit region^65^. Therefore, radiation induced ROS signaling may locate upstream of AP-1, MafB genes, which is an intriguing possibility.

Radiation induced responses include alteration of gene expression regulating immune reactions^42^. Induction of several regulatory gene including early response genes, cytokines are involved in transcriptional and subsequent pathological changes induced by radiation^67^. Cytokines genes including IL4 and IL22 are reported as induced after irradiation and aging which are expressed in thymocytes^17,68^. Such cytokines play roles for thymic regeneration including TECs ^69^. Regulation of IL4 was also discussed in view of radiation and its induction was classified as regulatory for anti-inflammatory cytokine response^70^. However, the roles of thymic mesenchyme and the possible interactions with TECs by such cytokines have not been studied extensively. In addition to the observation of radiation induced MafB gene, radiation induced IL4 gene expression was reported in this study. Of note is its subsequent reduction suggesting the transient and early phase of IL4 functions by such radiation. Currently, whether such induction is mediated by further upstream radiation induced genes or other relayed events are not known. Thymic mesenchyme constitutes rather minor population compared with the massive portion of TECs and thymocytes inside the organ. Effect of thymic mesenchyme might be also crucial regulating some aspects of TEC differentiation such as early progenitors which express FoxN1 and Krt5. By such putative regulation, abnormal TECs interaction with thymocytes may be triggered in MafB mutants after irradiation. IL4 signal may offer an intriguing research possibility for further investigations.

### MafB expression in thymic mesenchyme and its possible functions

Previous studies have shown that MafB is expressed in macrophages and monocytes of the adult mouse including the peritoneum, spleen, bone marrow and adipose tissues^32,40,62,71–73^. Part of target genes of MafB have been suggested as mouse scavenger receptor1 (MSR1) and macrophage receptor. However, its expression in the thymus has only been analyzed in mesenchyme by previous reports, and not in macrophages/monocytes. The present study revealed that a substantial portion of MafB-expressing cells in the adult thymus were F4/80 + macrophages/monocytes. Furthermore, a subpopulation of MafB-expressing cells was identified to be ER-TR7 + perivascular fibroblasts^37,43,74–76^. Altogether, the identification of these MafB-expressing cell types may offer some cues for the observed thymic defects.

Intriguingly, a recent study demonstrated that MafB-deficient macrophages possess a reduced capacity to engulf apoptotic cells and that MafB-deficient mice show increased susceptibility to autoimmune-inducing conditions, such as irradiation^62^. Because such study only examined macrophages derived from the bone marrow, spleen and peritoneum, it remains to be seen whether MafB-deficient thymic macrophages exhibit similar defects.

Macrophages/monocytes and fibroblasts perform various functions in the adult thymus, and many of these functions are known to closely associate with thymic vasculature^45,74,77,78^. Many MafB-expressing cells were shown to localize adjacent to thymic perivascular regions. These findings suggest possible roles of MafB in supporting thymic vasculature, such as through expression of growth factors, structural support and/or barrier functions^45,77–80^. Previous studies have established that thymic vascular endothelial cells are crucial for thymic regeneration^18^. Hence, it may be possible that MafB-expressing perivascular cells around PCVs are also involved in such process^45,74,81,82^.

### Thymic regeneration after sublethal total body irradiation (SL-TBI) and the roles of mesenchymal MafB gene

During thymic recovery after SL-TBI, *MafB*^+*/GFP*^ mice displayed impaired restoration of thymic medullary complexity and mTEC cluster organization compared to WT counterparts. Medullary regions correspond to the branches and detached parts of an intricate medullary network inside the thymus^14,47^. Medullary complexity (approximate number of region per lobe) thus represents an estimate of the number and organization of branches/islets in the entire medulla region^28^. A high degree of medullary complexity is a major feature of normal thymic architecture necessary for efficient thymocyte development^14,24,25^. Previous studies have also shown that medullary complexity (including mTEC clusters) is partially reduced by total body irradiation, but is subsequently restored during thymic recovery in young and healthy mice^14^. In addition to the prominent defects in thymic size and total thymocyte number, the current study revealed impaired restoration of thymic architecture in *MafB*^+*/GFP*^ mice after SL-TBI, judged by the reduced number of medullary region and aberrant mTEC organization. Previous studies have suggested that such reduction in medullary complexity decreases the overall efficiency of thymocyte development^14^. These perturbations in the epithelial compartment can affect the availability of microenvironmental niches and stromal signals involved in thymocyte development see below^52,83^.

### The status of TECs in the MafB mutants and Krt5 and FoxN1 as possible markers.

The current abnormality of medullary architecture is further supported by the observation that Keratin 5/8 double positive cells were less prominently detected in the cortex of *MafB*^+*/GFP*^ thymi, 7 days after SL-TBI. Such double positive cells have been reported to include thymic epithelial progenitor cells (TEPC) regulating the development and organization of mTECs^46,48–53,84^. Cortical Keratin 5/8 double positive cells were less prominently detected in *MafB*^+*/GFP*^ mice 7 days after SL-TBI (Fig. 4), reduced medullary complexity and aberrant organization of mTEC clusters were only observed 28 days after SL-TBI (Figs. 2, 3; and data not shown). Thus, the defects in Keratin 5/8 double positive cells occurred first and may have caused the subsequent reduction in medullary complexity (including mTEC clusters). On the other hand, no significant reduction in total thymocyte number was detected in *MafB*^+*/GFP*^ thymi compared to WT counterparts 7 days after SL-TBI (data not shown), whereas prominent reduction in total thymocyte number was observed in mutant mice compared to WT counterparts 28 days after SL-TBI (Fig. 1). Therefore, such epithelial defects preceded the defects in total thymocyte number.

It is well known that FoxN1, the causative gene for the nude mice, is one of the central regulators for thymic development^85^. Postnatal FoxN1 plays essential role for the maintenance of cTEC and mTEC. Augmented FoxN1 expression is shown by TBI treatment which plays essential role for thymic regeneration. In the current study, reduced FoxN1 gene expression was detected in *MafB*^+*/GFP*^ thymus. In addition, another immature TECs marker Krt5 expression was relatively lower compared with controls. In contrast, mature TECs marker CD40 gene expression shows similar expression level. These results may imply that *MafB*^+*/GFP*^ mutant TECs number reduced albeit the mature TECs population remains significantly. Thymic mesenchymal cells constitute rather minor cell populations compared with thymocytes and TECs. In fact, a few of reports showed thymic mesenchymal derived abnormalities such as recovery including the case for radiation. It has been shown that abnormal medullary structures associated with reduced number of immature TECs are one of the major thymic environmental changes. Because MafB is expressed in thymic mesenchyme, such effects could be relayed by interactions with TECs. It is plausible that impaired restoration of thymic medullary architecture may cause the defects in thymus size and total thymocyte number possibly through TECs. Further studies are necessary to clarify TECs abnormalities in *MafB*^+*/GFP*^ mutant.

### Radiation inducible MafB gene is involved for the regulation of thymic regeneration and aging

**Figure 8:**
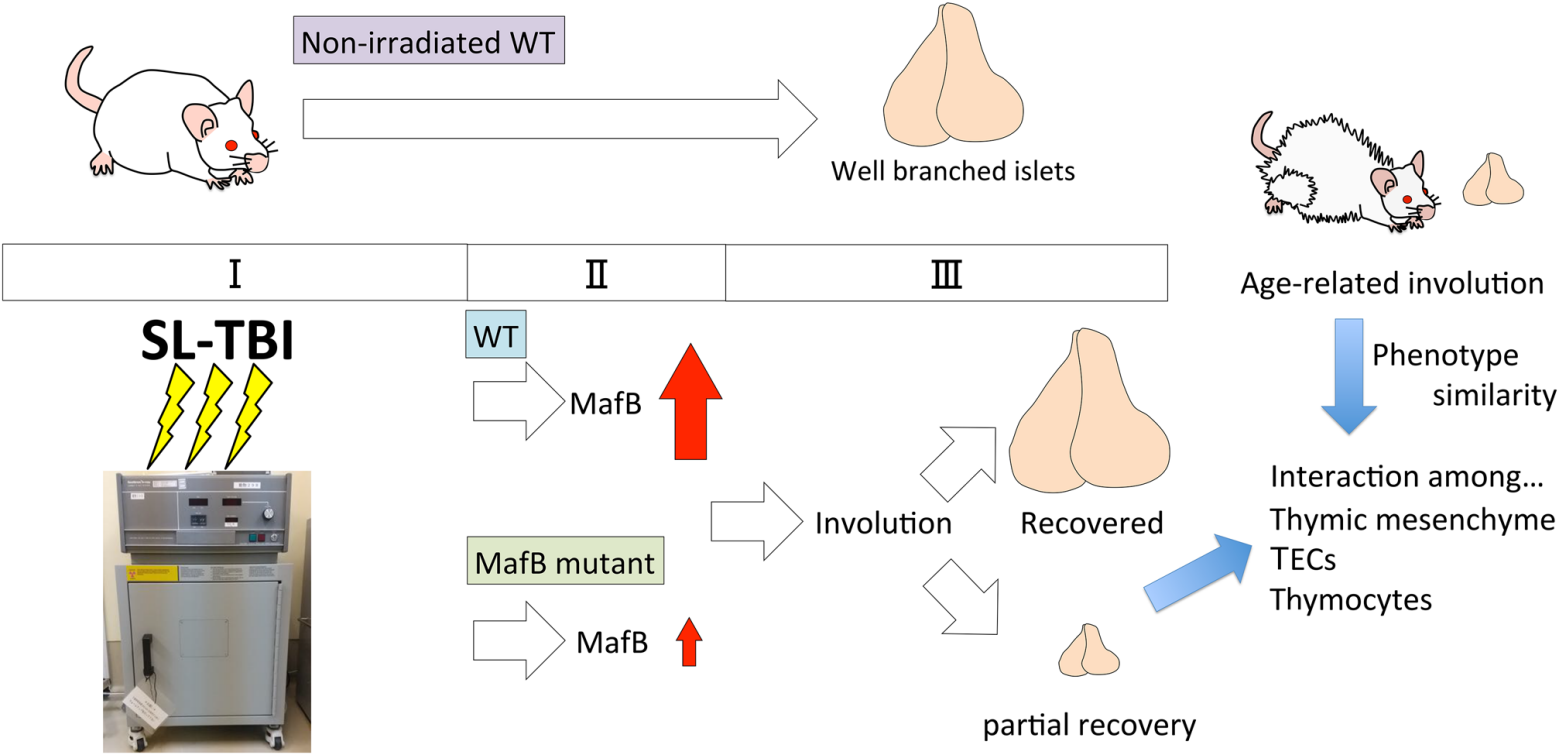
Implications of radiation induced MafB gene for thymic regeneration and aging. Summary of radiation (SL-TBI) induced MafB gene and its possible functions for thymic regeneration and age-related thymic involution. MafB is drastically induced at 1 day after irradiation. Its induction was decreased in *MafB*^+*/GFP*^ mutant. In the case of WT, thymus is recovered after 28 days irradiation. On the other hand, *MafB*^+*/GFP*^ mutant showed partial recovery including medullary region and epithelial abnormalities. Aged *MafB*^+*/GFP*^ mice thymi showed signs of accelerated thymic involution with altered islets structures. Possible effects of MafB mutation on thymic cellular interaction; Mesenchyme derived regulation for TECs population could lead to change of the immature TECs marker expression such as FoxN1 and Krt5 which is evident after thymic irradiation. Such cellular changes in TECs population may affect to the thymic involution and thymocyte development.

The current results and schema are summarized in Fig. 8. Figure 8 shows the impaired thymic recovery phenotypes of MafB mutants. WT and MafB mutants were irradiated by SL-TBI (Fig. 8 I). Prominent MafB gene induction was observed after SL-TBI in WT (Fig. 8II). Of note is the induction of IL4, one of the inducible cytokines necessary for recovery of some tissue damages was detected in such SL-TBI of WT. Such induction of MafB and IL4 was not prominent in case of MafB mutants suggesting their essential roles during SL-TBI. In case of WT, thymic recovery was observed after SL-TBI, while only partial recovery was observed in case of irradiated MafB mutants (Fig. 8III).

Age-related thymic involution is widely known as one of the important age-related thymic phenotypes. Significant histological abnormalities including islet complexity was shown as MafB mutant phenotypes. Possible contribution of thymic medullary region and alteration of TEC marker genes for impaired thymic recovery were suggested. It has been known that thymic mesenchyme and TECs versus thymocytes, which comprise major population in the thymus, play important roles during thymic regeneration. Stroma functions related with TEC function should be further investigated.

## Materials and methods

### Mice

The *mafB*/green fluorescent protein (GFP) knock-in null heterozygous mutant mouse strain (*MafB*^+*/*GFP^) was described previously^40^. In such mice, the GFP gene has been inserted into the *mafB* locus by homologous recombination. This target gene modification results in the introduction of loss-of-function mutation in *mafB*, and GFP expression within MafB-expressing cells. The mutant mice (*MafB*^+*/*GFP^) survived into adulthood without showing obvious abnormalities. Both of WT and mutant male mice were irradiated at 5 weeks olds. Their thymi were analyzed at 1 day, 7 days (6 weeks olds), 14 days (7 weeks olds), 21 days (8 weeks olds) and 28 days (9 weeks olds) after irradiation. The mice were maintained in a mixed genetic background of C57BL/6 J and ICR strains. All experimental procedures were performed in accordance with the guidelines of Wakayama Medical University Animal Care and Use Committee (Approval number: 732). Moreover, all procedures involving genetically modified mice were also approved by the same committee (Approval number: 28–9). The study was carried out in compliance with the ARRIVE guidelines.

### Quantitative reverse transcription-PCR (qRT-PCR) analysis

Whole thymi of 5-week-old wild-type (WT) and *MafB*^+*/GFP*^ male littermates or irradiated thyme at each timepoints were excised, and total RNA was extracted using an ISOGEN II kit (Nippon Gene, Toyama, Japan). RNA purity and yield were determined with a Nanodrop spectrophotometer (Nanodrop Technologies). To generate complementary DNA (cDNA) from total RNA, reverse transcription-PCR (RT-PCR) was achieved using the PrimeScript RT Master Mix (Perfect Real Time) (Takara Bio, Otsu, Japan) and the DNA Engine Peltier Thermal Cycler (Bio-Rad). Subsequently, quantitative real-time PCR (qPCR) was performed in the StepOnePlus Real-Time PCR System (Applied Biosystems, Foster City, CA) using the SYBR Premix Ex Taq II (Tli RNaseH Plus) (Takara Bio, Otsu, Japan), all according to the manufacturer’s instructions. The mRNA levels of genes were quantitatively measured and normalized to those of *gapdh* (glyceraldehyde-3-phosphate dehydrogenase [GAPDH]), using the relative standard curve method^86^.

The following primer sequences were used:

- *mafB* forward, 5′- TTCGACGTGAAGAAGGAGCC-3′.
- *mafB* reverse, 5′- AAGCTGGGAGAAGAAGGCAC-3′.
- *gapdh* forward, 5′- AACGACCCCTTCATTGACCTC-3′.
- *gapdh* reverse, 5′-CCTTGACTGTGCCGTTGAATT-3′.
- *IL4* forward, 5′-GATGGATGTGCCAAACGTCC-3′.
- *IL4* reverse, 5′-CTTGGAAGCCCTACAGACGA-3′.
- *Krt5* forward, 5′-AAGATGTTCTTTGATGCGGAGC-3′.
- *Krt5* reverse, 5′-TCCATGGAAAGGACCACAGATG-3′.
- *FoxN1* forward, 5′-CCAGCATCGCATCTCCAGAC-3′.
- *FoxN1* reverse, 5′-TTTCTGAAGGAGAAAGGCGGAA-3′.
- *CD40* forward, 5′-TTGTTGACAGCGGTCCATCT-3′.
- *CD40* reverse, 5′-TTCCTGGCTGGCACAAATCA-3′.

## Thymic gross morphology and cellularity (total thymocyte number) analysis

Male mice were euthanized by cervical dislocation at 9 weeks of age, then weighed. Thymi were subsequently excised, placed in ice-cold 1 × PBS, and weighed. Images of thymic gross morphology were acquired using a stereo microscope (Leica M165 FC). Cells were then harvested by gently grinding each thymus between two frosted glass slides. After thoroughly grinding each thymus, the resulting cell suspension was passed through a filter (42 µm pore size) into a 50-mL conical centrifuge tube. Thymocytes were pelleted out of the cell suspension by centrifugation (TOMY refrigerated centrifuge LX-120) at 1000 rpm for 7 min, then washed and re-suspended in fresh ice-cold 1 × PBS. To determine total thymocyte number, the cells were counted using a haemocytometer (ERMA, Tokyo; 0490). Trypan blue exclusion method was used during cell counting to distinguish dead cells from viable cells.

### Sublethal total body irradiation (SL-TBI) model of thymic damage

Prior to irradiation, anesthetic solution (medetomidine hydrochloride 0.03 mg/ml, midazolam 0.4 mg/ml, and butorphanol tartrate 0.5 mg/ml) was administered intraperitoneally to 5-week-old male mice, at a dose of 0.01 mL/g of body mass. After complete anesthetization, mice were exposed to total body radiation for 11 min and 28 s (sublethal irradiation dose of 5.5 Gy) using an X-Radiator RX-650 (Faxitron) (voltage setting at 90 kVP)^15,17^. After 1, 7, 14, 21, and 28 days of recovery, SL-TBI-treated mice and untreated littermates were euthanized by cervical dislocation and weighed. Thymi were excised then analyzed as above, or prepared for histological and q-PCR analyses.

### Hematoxylin and Eosin (H.E.) staining and microscopy

Excised thymi were fixed overnight at 4 °C in 4% (wt/vol) paraformaldehyde (PFA) dissolved in PBS. These tissues were subsequently dehydrated in methanol, paraffinized, and embedded in paraffin. Transverse Sects. (6-μm thick) and sagittal Sects. (6-μm thick) were prepared from the thymi. H.E. staining was performed by standard procedures as previously described^87^. Images were acquired using the bright-field view of a standard microscope (OLYMPUS BX51, DP80).

To quantify thymic medullary complexity, the number of medullary regions per lobe was estimated based on a method used in previous studies^28^. The images of all transverse paraffin sections of each thymus lobe (from the anterior to posterior levels) were examined individually, and the number of distinct medullary region (medullary areas circumscribed by cortical area) per section were counted every 5 ~ 10 sections. This method of analysis resulted in the counting of 683 thymic Sects. (358 WT sections and 325 *MafB*^+*/*GFP^ sections) out of a total of 4,323 sections from the following pairs of SL-TBI-treated littermates: (1) 79 out of a total of 788 WT thymic sections, and 64 out of a total of 638 *MafB*^+*/*GFP^ thymic sections from the first pair of littermates. (2) 139 out of 724 WT, and 137 out of 704 *MafB*^+*/*GFP^ thymic sections from the second pair. (3) 140 out of 783 WT, and 124 out of 686 *MafB*^+*/*GFP^ thymic sections from the third pair. The bars in the resulting bar graph represented the mean ± standard deviation for 358 tissue sections from three WT mice, and 325 tissue sections from three *MafB*^+*/*GFP^ mice.

### Immunofluorescence staining and microscopy

All thymi analyzed by fluorescence microscopy were taken from 6-to-12-week-old male mice. Excised thymi were either directly embedded in OCT compound (Sakura Tissue-Tek), or fixed overnight at 4 °C in 4% (wt/vol) PFA dissolved in PBTx (0.1% Triton X-100 in 1 × PBS).

For thymi directly embedded in OCT compound (Sakura Tissue-Tek), frozen Sects. (10-µm thick) were prepared using a cryostat (Microm HM 505 N). Prior to antibody incubation, frozen sections were fixed in acetone, washed with 1 × PBS, and blocked with 1% fetal bovine serum (FBS) in PBS for 1 h at room temperature (RT) in a moisture box. Primary antibody staining was also performed at RT for 1 h in a moisture box, and the following antibodies were utilized (with indicated dilutions, codes, clones, and sources): rabbit anti-Keratin 5 (1:100, PRB-160P, BioLegend), mouse anti-Keratin 8 (1:100, 61,038, PROGEN), rabbit anti-Keratin 14 (1:100, PRB-155P, BioLegend), and rat ER-TR7 (1:100, T-2109; BMA Biomedicals). After the sections were washed twice with PBS, secondary antibody staining was performed at RT for 1 h in a dark moisture box, and the following antibodies and dilutions were utilized: anti-mouse goat IgG Alexa-488 (1:200), anti-rabbit goat IgG Alexa-546 (1:200), and anti-rat goat IgG Alexa-488 (1:200) (all antibodies from Molecular Probes; Life Technologies).

For thymi that were fixed overnight at 4 °C in 4% (wt/vol) PFA dissolved in PBTx (0.1% Triton X-100 in 1 × PBS). Fixed thymi were washed multiple times in PBTx, then coursed through a sucrose gradient series (10% sucrose in 1 × PBS, 15%, 20%, 20% OCT [Sakura Tissue-Tek] 1:1 overnight at 4 °C). Samples were then embedded in OCT at -80 °C, followed by sectioning at 20 um thickness per section using a cryostat (Microm HM 505 N). The resulting frozen section slides were dried on a slide warmer at 37 °C for 30 min followed by multiple washes with blocking solution (10% FBS, 3% BSA, dissolved in PBTx). After washing and incubating the slides in blocking solution for 1 h, these were incubated overnight in primary antibody solution (diluted in blocking solution) at 4 °C. The following primary antibodies were utilized (with indicated dilutions, codes, clones, and sources): rabbit anti-GFP (1:500, ab6556; Abcam), rat ER-TR7 (1:100, T-2109; BMA Biomedicals), rat anti-mouse F4/80 (1:400, MCA497GA, CI:A3-1; AbD Serotec), and rat anti-mouse CD31 (also known as PECAM-1; 1:400, 553,370, MEC 13.3; BD Pharmingen). After washing multiple times with washing solution (1% FBS, 3% BSA, dissolved in PBTx), the slides were incubated in blocking solution for another hour. Subsequently, slides were incubated in secondary antibody solution (diluted in blocking solution) under dark conditions for 1 h at room temperature. Alexa-488- and Alexa-546-conjugated secondary antibodies (Molecular Probes; Life Technologies) were both utilized at 1:200 dilution.

After final washing, samples were mounted with PermaFluor aqueous mounting medium (Thermo Scientific). All images were acquired using either a standard fluorescence microscope (OLYMPUS BX51), or a confocal laser scanning microscope (ZEISS LSM 700).

### Statistical analysis

All data were presented as the mean ± standard deviation (SD). Comparative statistical analysis was performed using the two-tailed Student’s *t* test or Tukey–Kramer methods, wherein *P*-values less than 0.05 were considered significant. Calculations were done using Excel software (Microsoft, Redmond, WA, USA).

## Supplementary Information

Supplementary Information 1.

Supplementary Information 2.

Supplementary Information 3.

Supplementary Information 4.

Supplementary Information 5.
