# Supplementary material for: Radiation inducible MafB gene is required for thymic regeneration
Source: Sci Rep. 2021 May 17;11:10439. doi: 10.1038/s41598-021-89836-7 (PMC8129107; doi:10.1038/s41598-021-89836-7)
Supplement: Supplementary file 1 — Supplementary Information 1. [file 41598_2021_89836_MOESM1_ESM.docx]

**Radiation inducible MafB gene is required for thymic regeneration**

Daiki Hashimoto^1*^, Jose Gabriel R. Colet^1,2*^, Aki Murashima^3**^, Kota Fujimoto^1^, Yuko Ueda^4^, Kentaro Suzuki^1^, Taiju Hyuga^1^, Hiroaki Hemmi^5^, Tsuneyasu Kaisho^6^, Satoru Takahashi^7^, Yousuke Takahama^8^ and Gen Yamada^1**^

1. Department of Developmental Genetics, Institute of Advanced Medicine, Wakayama Medical University (WMU), Wakayama, Japan

2. Experimental Therapeutics Laboratory, University of South Australia Cancer Research Institute, Clinical and Health Sciences, University of South Australia, Adelaide, SA, Australia

3. Department of Anatomy, Iwate Medical University, Yahaba, Iwate, Japan

4. Department of Urology, Wakayama Medical University, Wakayama, Japan

5. Laboratory of Immunology, Faculty of Veterinary Medicine, Okayama University of Science, Imabari, Ehime, Japan

6. Department of Immunology, Institute of Advanced Medicine, Wakayama Medical University, Kimiidera, Wakayama, Japan

7. Department of Anatomy and Embryology, Faculty of Medicine, University of Tsukuba, Tennodai, Japan

8. Experimental Immunology Branch, National Cancer Institute, NIH, Bethesda, MD 20892, USA.

*** equal contribution**

**** corresponding author**

Daiki Hashimoto

Department of Developmental Genetics, Institute of Advanced Medicine, Wakayama Medical University (WMU), Wakayama, Japan. hashi213@wakayama-med.ac.jp

Jose Gabriel R. Colet

Department of Developmental Genetics, Institute of Advanced Medicine, Wakayama Medical University (WMU), Wakayama, Japan

Experimental Therapeutics Laboratory, University of South Australia Cancer Research Institute, Clinical and Health Sciences, University of South Australia, Adelaide, SA, Australia. josegabrielcolet@gmail.com

Aki Murashima

** corresponding author

Department of Anatomy, Iwate Medical University, Yahaba, Iwate, Japan. amura@iwate-med.ac.jp

Kota Fujimoto

Department of Developmental Genetics, Institute of Advanced Medicine, Wakayama Medical University (WMU), Wakayama, Japan. kfujimot@outlook.jp

Yuko Ueda

Department of Urology, Wakayama Medical University, Wakayama, Japan. yuko0519@wakayama-med.ac.jp

Kentaro Suzuki

Department of Developmental Genetics, Institute of Advanced Medicine, Wakayama Medical University (WMU), Wakayama, Japan. k-suzuki@wakayama-med.ac.jp

Taiju Hyuga

Department of Developmental Genetics, Institute of Advanced Medicine, Wakayama Medical University (WMU), Wakayama, Japan. hyuga520@wakayama-med.ac.jp

Hiroaki Hemmi

Laboratory of Immunology, Faculty of Veterinary Medicine, Okayama University of Science, Imabari, Ehime, Japan. h-hemmi@vet.ous.ac.jp

Tsuneyasu Kaisho

Department of Immunology, Institute of Advanced Medicine, Wakayama Medical University, Kimiidera, Wakayama, Japan. tkaisho@wakayama-med.ac.jp

Satoru Takahashi

Department of Anatomy and Embryology, Faculty of Medicine, University of Tsukuba, Tennodai, Japan. satoruta@md.tsukuba.ac.jp.

Yousuke Takahama

Experimental Immunology Branch, National Cancer Institute, NIH, Bethesda, MD 20892, USA. yousuke.takahama@nih.gov

Gen Yamada

**corresponding author

Department of Developmental Genetics, Wakayama Medical University

Kimiidera 811-1,Wakayama City, Wakayama 641-8509, Japan

Tel: 81-73-441-0849

Fax: 81-73-499-5026

genyama77@yahoo.co.jp

**Supplementary Figure legends**

**Supplementary Figure 1 MafB-expressing cells appeared to locate adjacent to CD31+ vascular endothelium.** (a-c) GFP expression (green color, representing MafB-expressing cells) and CD31 expression (red color, representing vascular endothelium) shown by immunofluorescence staining of *MafB^+/GFP^* thymus frozen sections (transverse). Low magnification images. White asterisks indicate postcapillary venules (vessel diameter greater than 15 µm).

**Supplementary Figure 2. Thymi of untreated *MafB^+/GFP^* mice appeared similar to those of wild-type littermates.** (a-c) Thymi from untreated 9-week-old wild-type (WT) (white bars, n=6) and *MafB^+/GFP^* (black bars, n=6) mice were analyzed. (a) Absolute thymus mass (mg; milligrams). (b) Normalized mass calculated as thymus mass divided by body mass (mg/g). (c) Total thymocyte number, estimated using a haemocytometer. All data presented as the means ± SD. *P < 0.05 (Student’s *t* test). All data shown are representative results of at least 3 independent experiments using adult specimens from different litters (n ≥ 3).

**Supplementary Figure 3. *MafB^+/GFP^* mice showed impaired restoration of thymic architecture after SL-TBI.** (a-d) Hematoxylin and Eosin (H.E.) staining of 9-week-old *MafB^+/GFP^* and WT thymi paraffin sections (sagittal), 28 days after SL-TBI. For each thymic lobe, multiple images of the same sagittal section were merged into a single large image to reveal the entire tissue area. The borders of medullary region are indicated by black dotted lines. Scale bar: 200 µm. Multiple medullary region were observed in (a) untreated WT thymi, (b) untreated *MafB^+/GFP^* thymi, and (c) SL-TBI-treated WT thymi. (d) SL-TBI-treated *MafB^+/GFP^* thymi showed reduced number of medullary region compared to those of SL-TBI-treated WT littermates. All data shown are representative results of 3 independent experiments using adult specimens from different litters (n=3).

**Supplementary Figure 4. Thymic mass was slightly reduced in 12-month-old *MafB^+/GFP^* mice compared to WT counterparts.** (a-b) Thymi from untreated 12-month-old WT (white bars, n=5) and *MafB^+/GFP^* (black bars, n=7) mice were analyzed. (a) Absolute thymus mass (mg; milligrams). (b) Normalized mass calculated as thymus mass divided by body mass (mg/g). All data presented as the means ± SD. *P < 0.05 (Student’s *t* test). All data shown are representative results of at least 3 independent experiments using adult specimens from different litters (n ≥ 3).
