# Supplementary figures and images for: Radiation inducible MafB gene is required for thymic regeneration
Source: Sci Rep. 2021 May 17;11:10439. doi: 10.1038/s41598-021-89836-7 (PMC8129107; doi:10.1038/s41598-021-89836-7)

**GFP (MafB)**

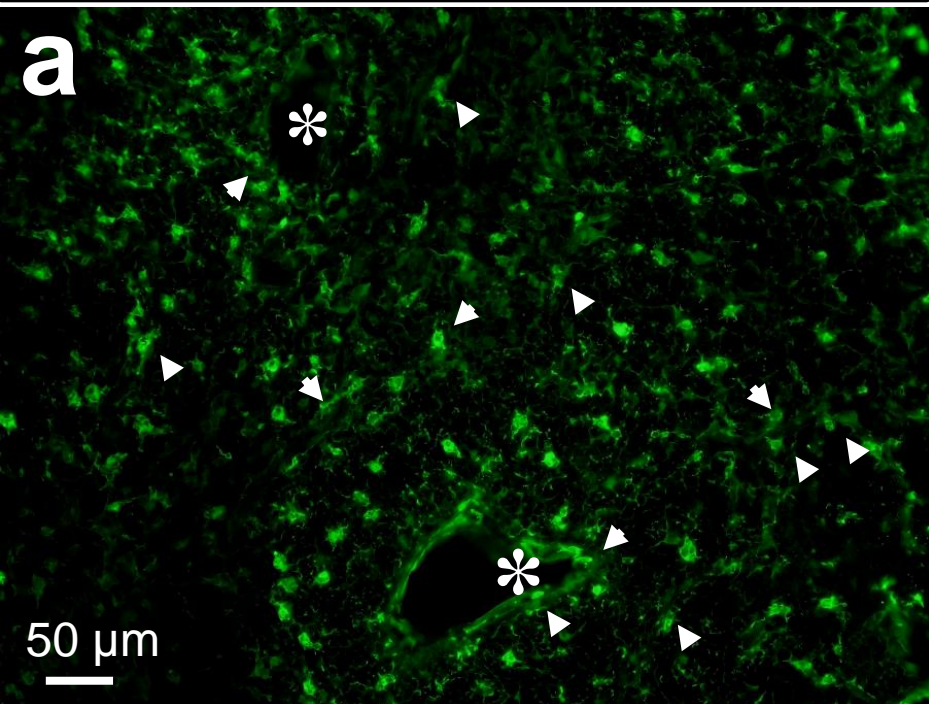

**CD31**

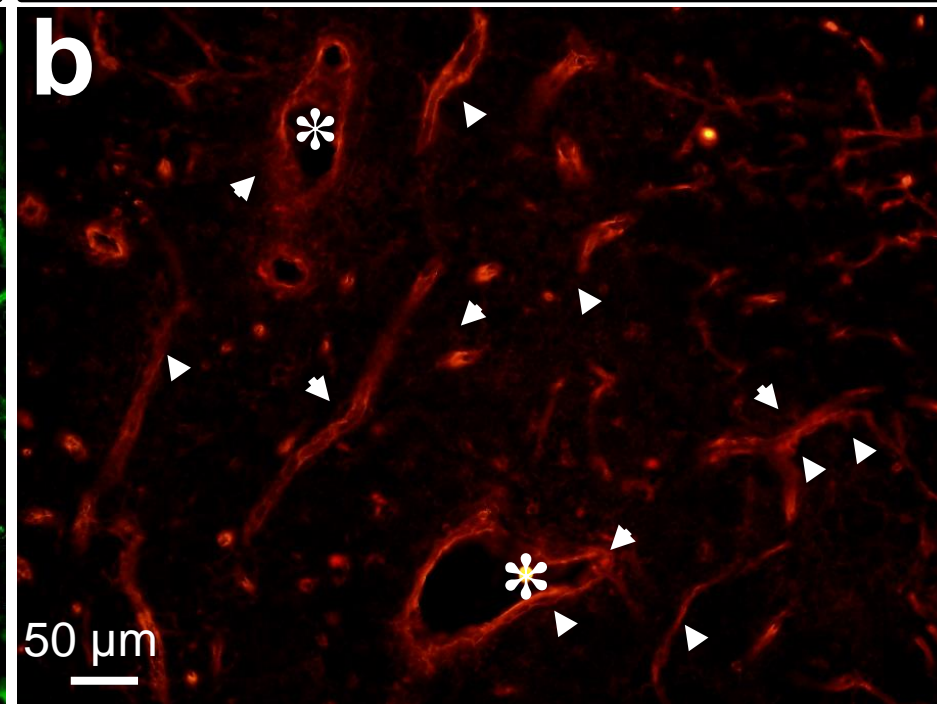

**GFP and CD31**

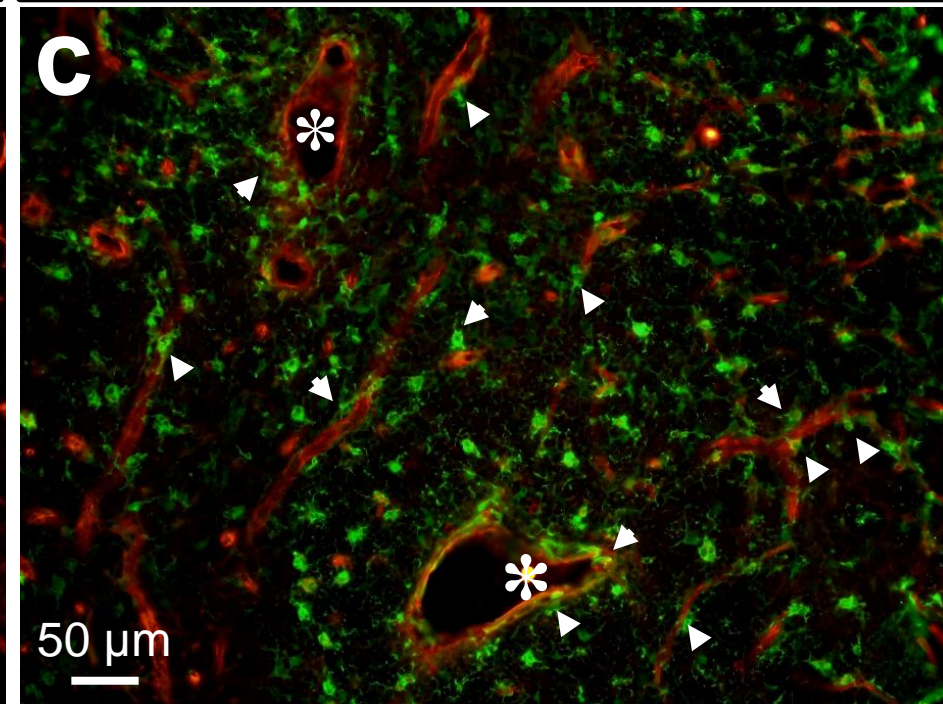

Supplement: Supplementary file 2 — Supplementary Information 2. [file 41598_2021_89836_MOESM2_ESM.pdf]

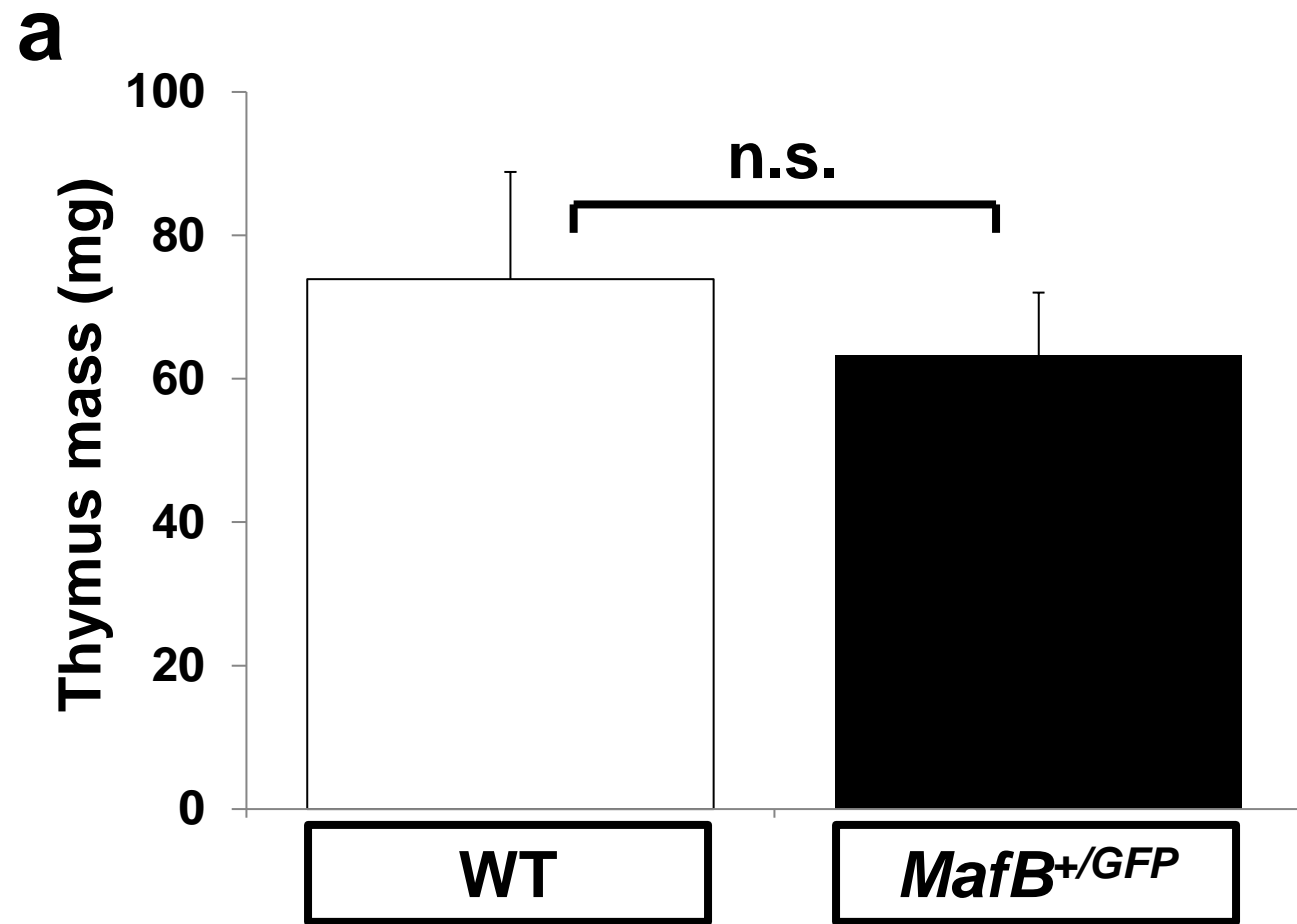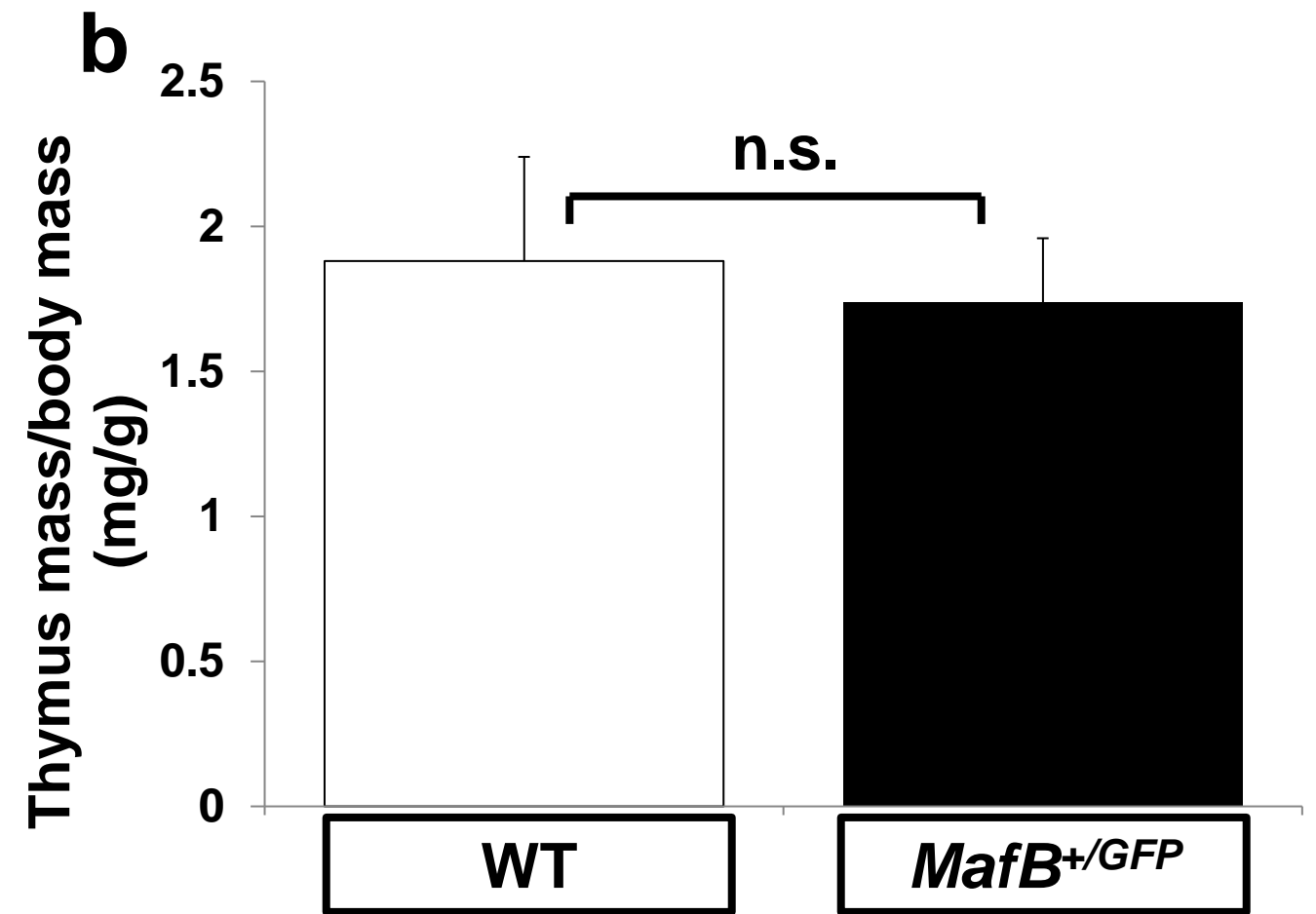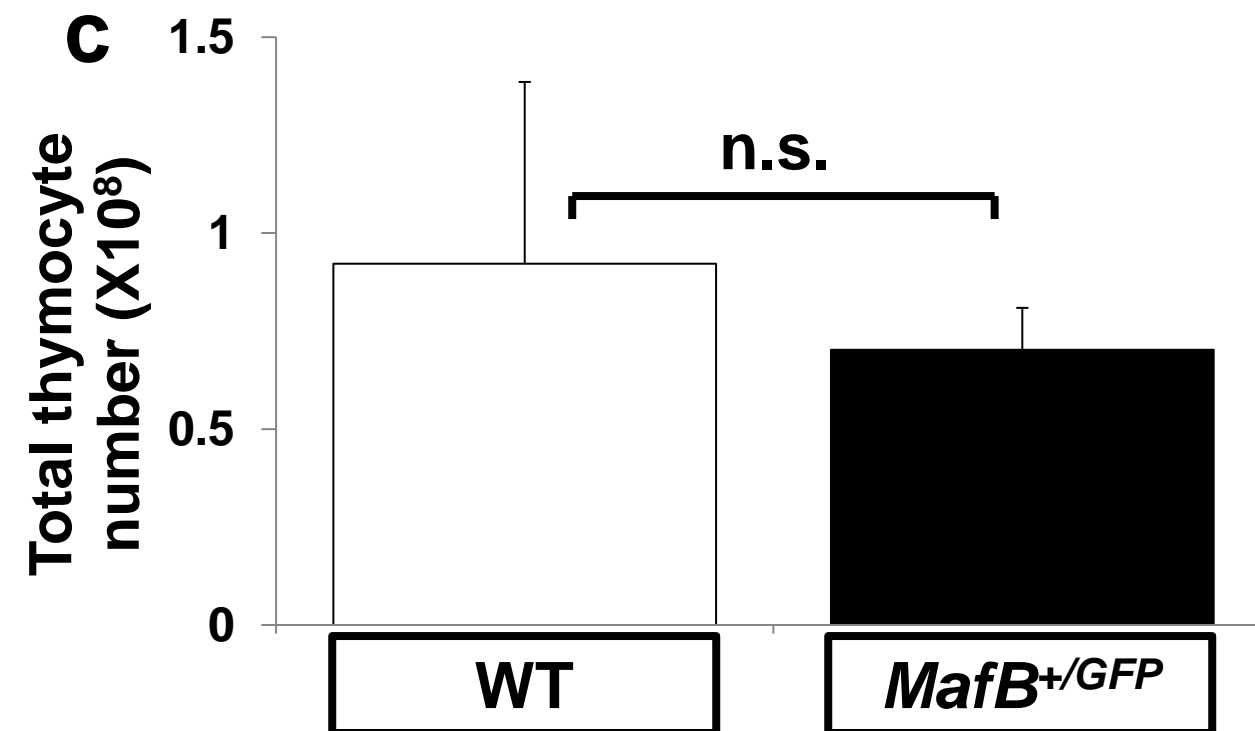

Supplement: Supplementary file 3 — Supplementary Information 3. [file 41598_2021_89836_MOESM3_ESM.pdf]

Untreated

SL-TBI

WT

a

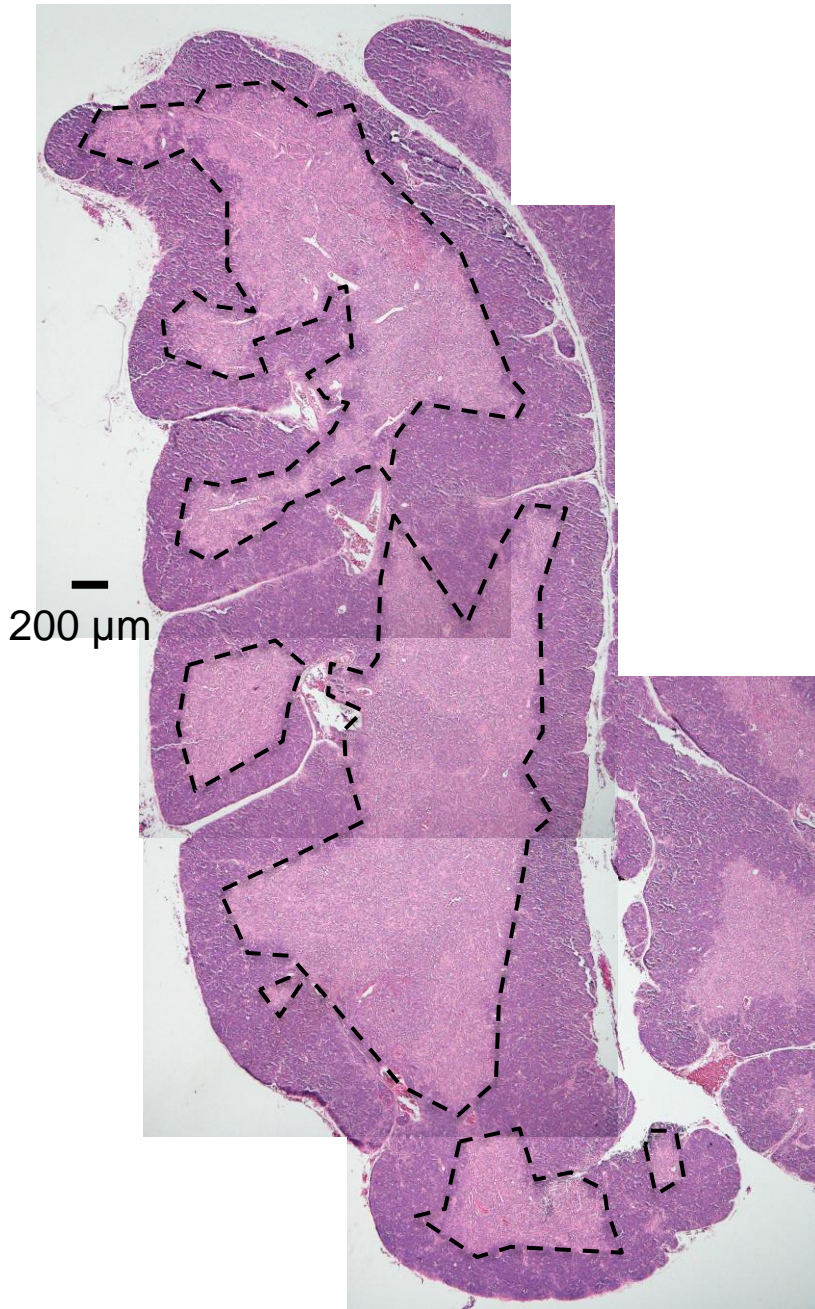

c

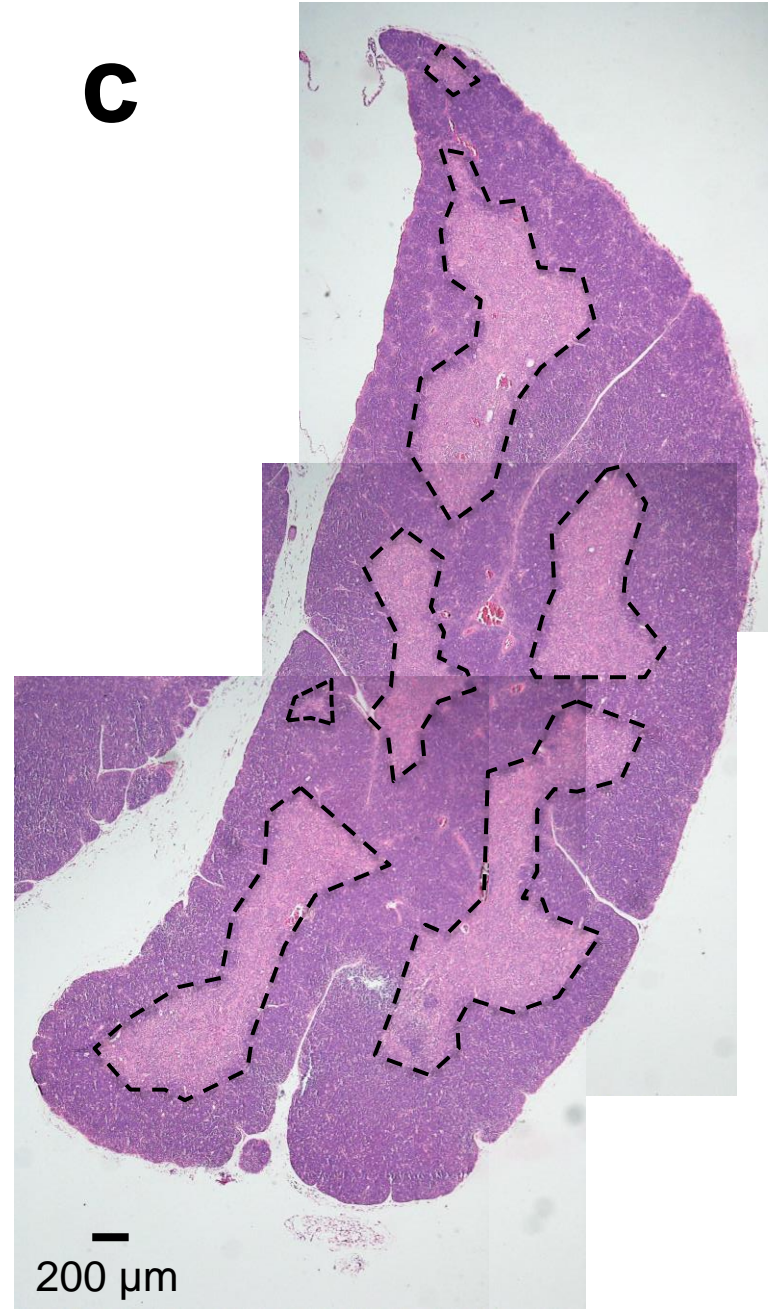

b

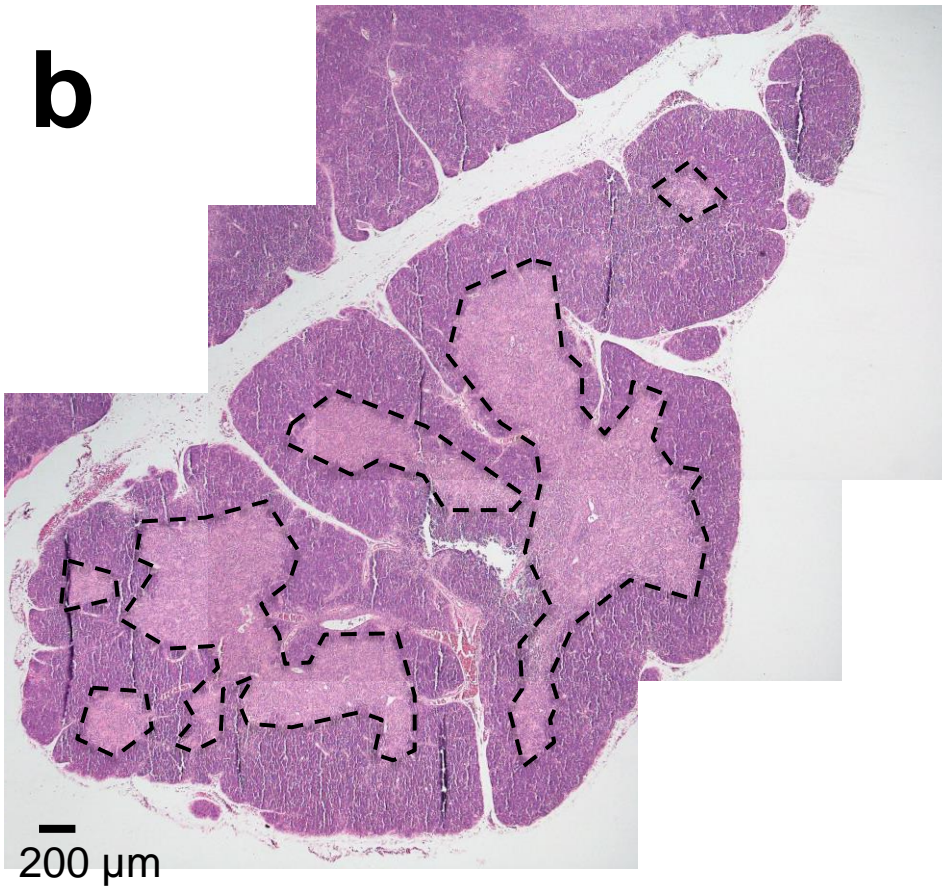

d

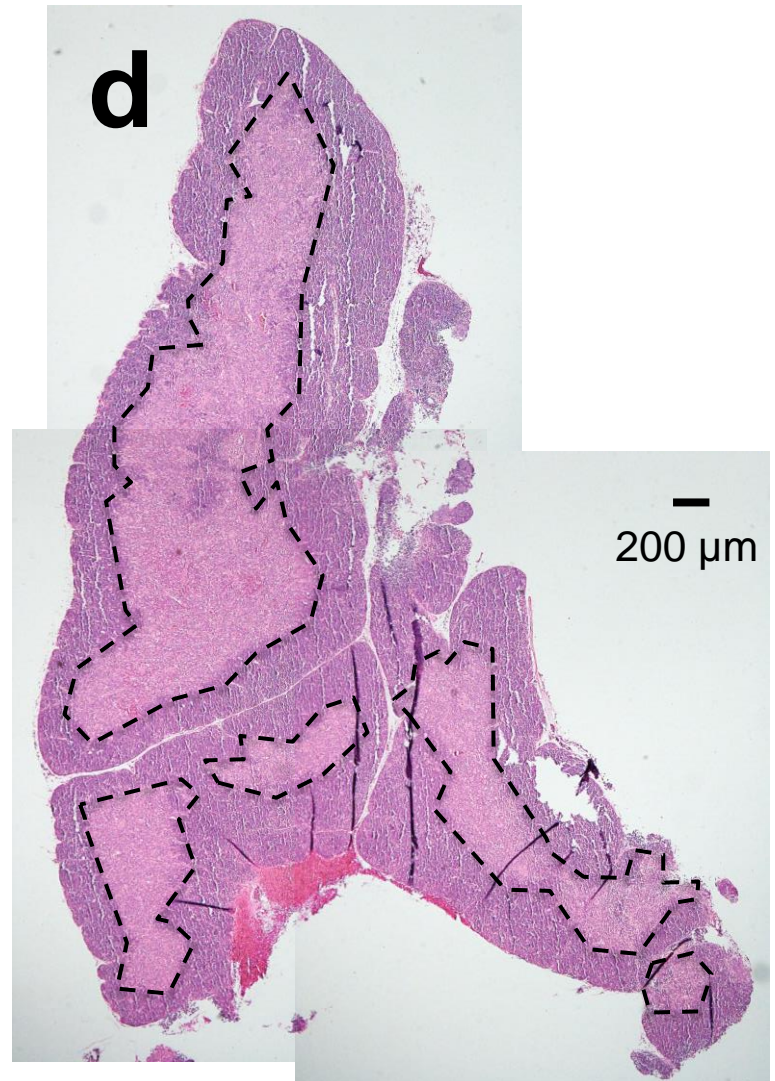

*MafB*<sup>+/GFP</sup>

Supplement: Supplementary file 4 — Supplementary Information 4. [file 41598_2021_89836_MOESM4_ESM.pdf]

**a**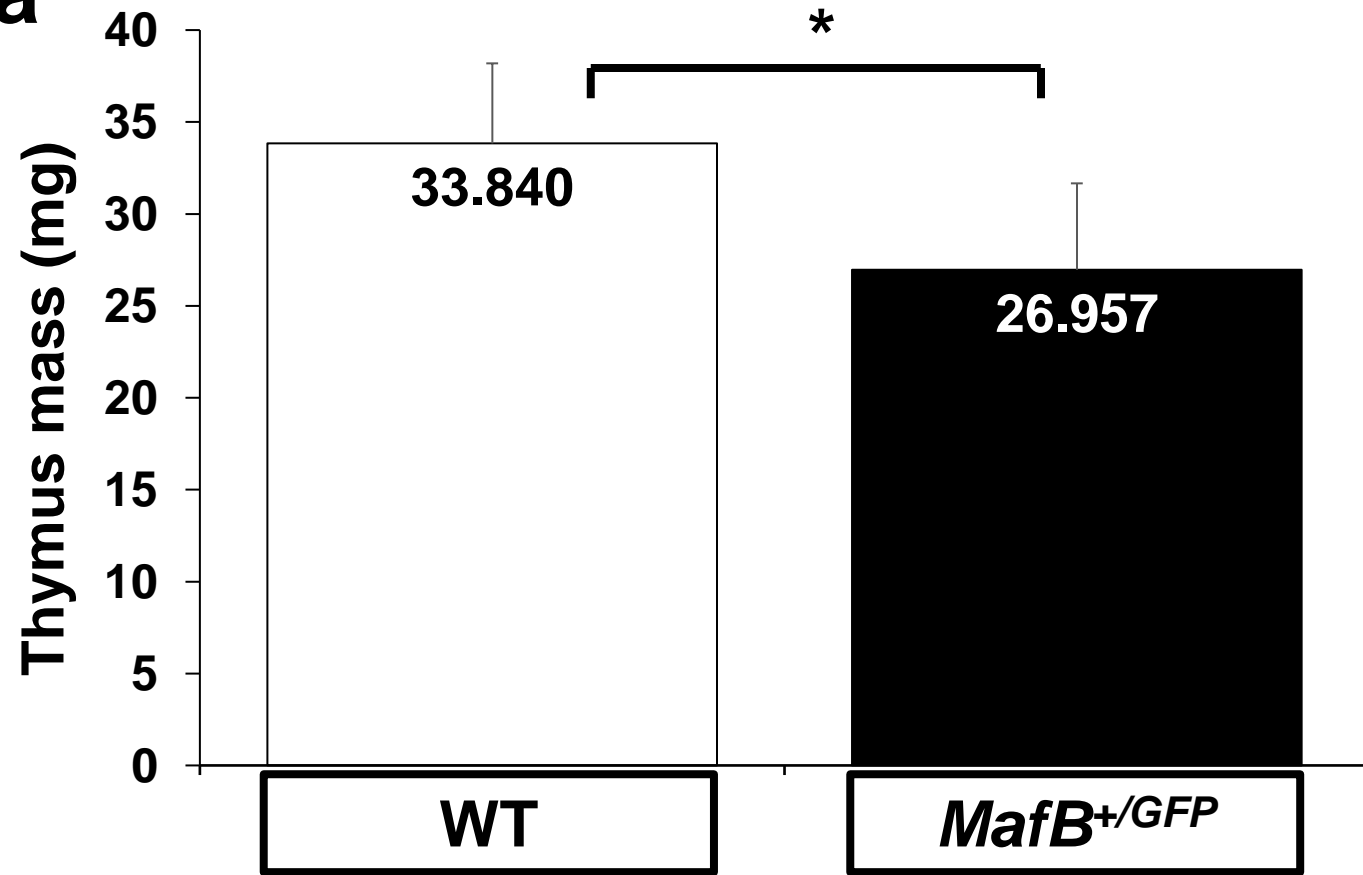**b**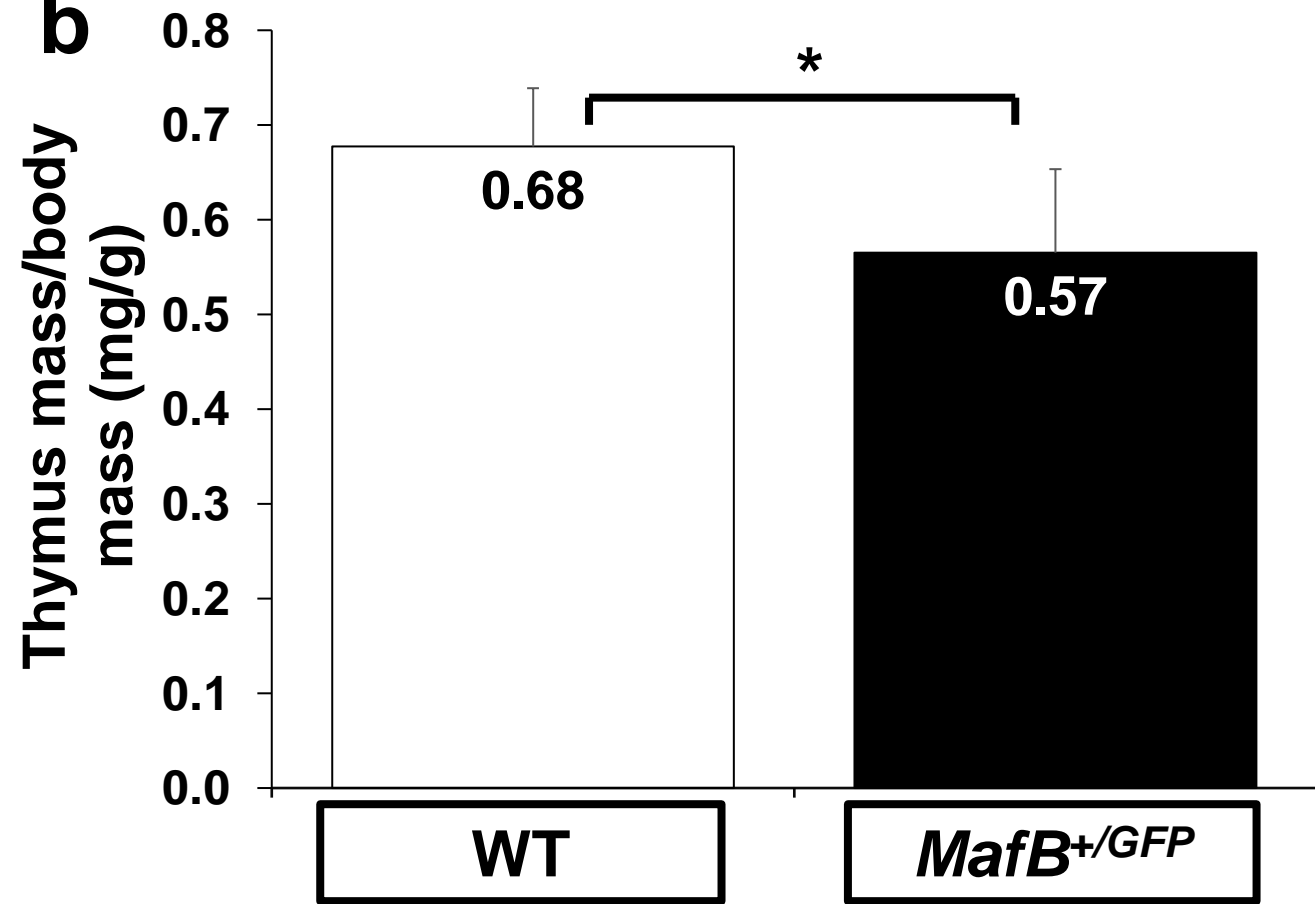

Supplement: Supplementary file 5 — Supplementary Information 5. [file 41598_2021_89836_MOESM5_ESM.pdf]
